# Harnessing Dynamic Electrostatic Fields for Energy Generation with Diode Cells

**DOI:** 10.1002/advs.202505476

**Published:** 2025-05-14

**Authors:** Renyun Zhang, Magnus Hummelgård, Ye Xu, Martin Olsen, Jonas Örtegren, Göran Thungström, Henrik Andersson, Zhong Lin Wang

**Affiliations:** ^1^ Department of Engineering, Mathematics, and Science Education Mid Sweden University Sundsvall SE 85170 Sweden; ^2^ Department of Computer and Electrical Engineering Mid Sweden University Sundsvall SE 85170 Sweden; ^3^ Beijing Institute of Nanoenergy and Nanosystems Chinese Academy of Sciences Beijing 100083 China; ^4^ School of Nanoscience and Technology University of Chinese Academy of Sciences Beijing 100049 China

**Keywords:** diode cells, electrostatic fields, energy harvesting, implanted sensors

## Abstract

Harvesting energy from distributed mechanical motions has garnered significance in future power sources for small electronics and sensors. Although technologies like triboelectric nanogenerators have shown promising results, their efficacy hinges on the alignment of motion vectors and device architectures. Here, an approach employing stationary diode cells (DiCes) to generate electricity is presented. This approach leverages dynamically changing electrostatic fields to induce potential differences across diode junctions via electrostatic induction, which is verified theoretically and experimentally. DiCes constructed with multiple diodes can directly output DC voltage and current. A 0.02 m^2^ sized DiCe contains 360 diodes can supply a DC voltage and current of maximum 490 V and 1.08 mA, respectively, which equals a DC power density of 26.5 W·m^−2^. Capable of functioning in both contact and non‐contact modes, DiCes offer versatile applications, from wirelessly powering implanted medical devices to harvesting energy from vehicles and roads.

## Graphical abstract

Stationary diode cells (DiCes) are fabricated to harvest energy from dynamically changing electrostatic fields by leveraging the unique current modulation properties of diodes. The DiCes can directly output DC voltage and current. Their applications range from energy harvesting and powering implanted devices to use in sensors.

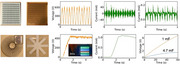

## 1 Introduction

Harvesting energy from distributed mechanical motions^[^ ^1^, ^2^ ^]^ holds significant importance in addressing the need for sustainable power sources,^[^ ^3^, ^4^ ^]^ particularly in scenarios where access to conventional power grids is limited, such as remote areas,^[^ ^5^ ^]^ wearable electronics,^[^ ^6^ ^]^ or implanted devices.^[^ ^7^ ^]^ Technologies with such purposes promote the lifespan of portable electronics and facilitate the development of self‐powered sensors.^[^ ^8^, ^9^ ^]^

Although the importance is significant, technology development remains challenging in feasibility, flexibility, and simplicity.^[^ ^10^, ^11^ ^]^ Triboelectric nanogenerators (TENGs)^[^ ^12^, ^13^, ^14^, ^15^ ^]^ and piezoelectric generators^[^ ^16^, ^17^ ^]^ are two technologies that have been widely studied to harvest energy from mechanical motions such as waves^[^ ^18^ ^]^ and to serve as high‐voltage resources for self‐powered systems such as autonomous actuation/digital microfluidic systems.^[^ ^19^ ^]^ However, devices based on the two technologies require the alignment of the direction of the motion vectors with the device architecture.

Here, we present a technology that utilizes the response of diodes to dynamically changing electrostatic fields for converting mechanical motions to electricity, namely diode cells (DiCes). A DiCe is a stationary device that guides electron flow across the junction upon the potential difference created by the changing electrostatic fields. The power density of a DiCe could achieve 362 kW·m^−3^ so that a single‐diode DiCe could power 305 LEDs. A 0.02 m^2^ sized DiCe contains 360 diodes can supply a DC voltage and current of maximum 490 V and 1.08 mA, respectively, which equals a DC power density of 26.5 W·m^−2^. Besides, the DiCes have unique features such as freedom in motion in space and on surfaces, as well as the volume effect that can boost the energy output. Such features allow the DiCes to be applied in many scenarios, such as powering implanted devices, and spontaneous energy harvesting from cars and roads. The development of DiCes opened a window for energy harvesting from distributed mechanical motions with flexibility, feasibility, and simplicity.

### 1.1 Theoretical Framework of Energy Harvesting in DiCes

**Figure 1:**
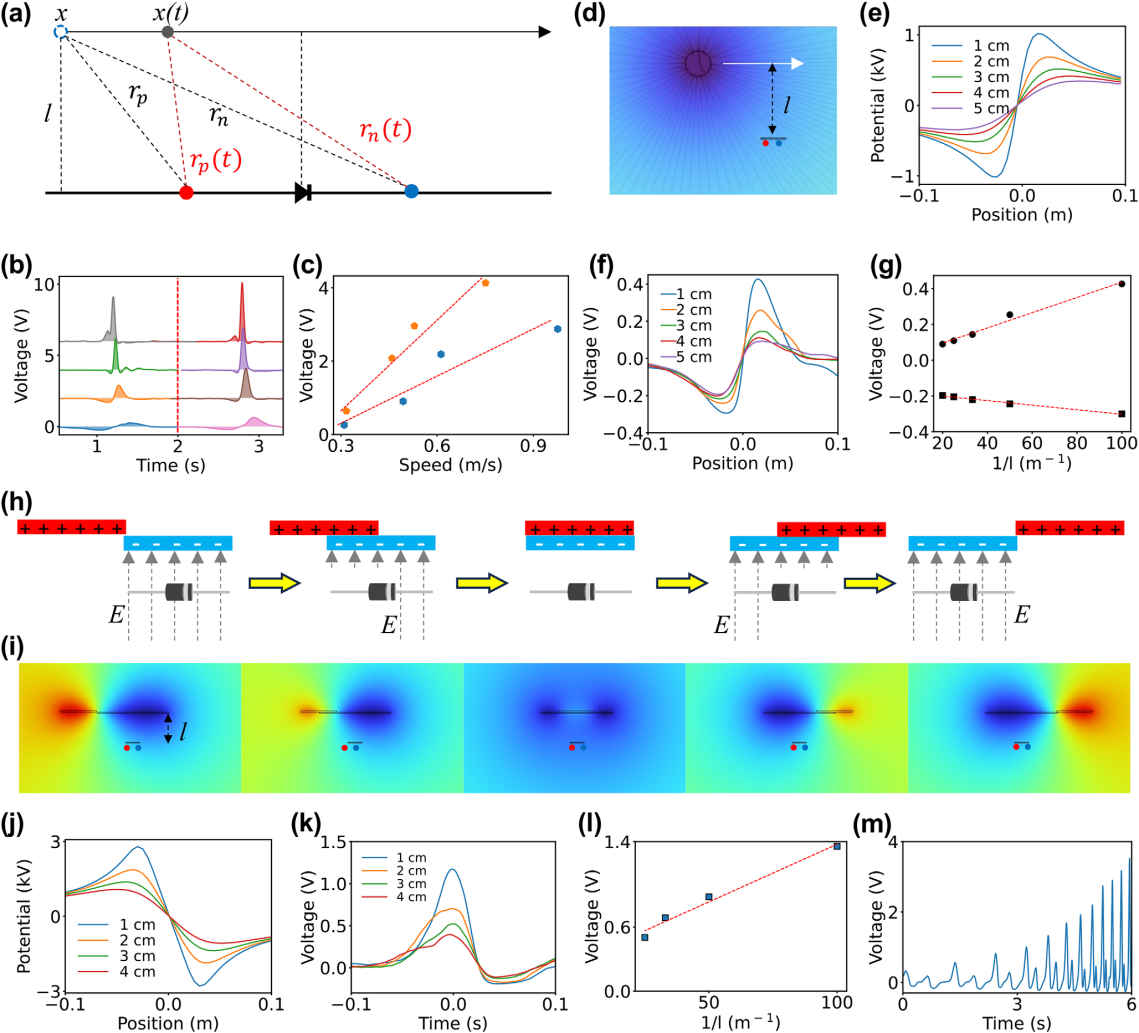
Electricity generation on DiCe. a) A sketch of the generation of electricity on a diode by moving a charged object over the diode. *l* is the vertical distance between the diode and the charged object. *x* is the lateral position of the charged object to the middle of the *pn* junction. *x(t)* is the lateral position at time *t*. *r_p_* and *r_n_* is the distance of the charged object to the *p* and *n* side of the diode, *r_p_(t)* and *r_n_(t)* is the distances of the charged object at time *t*. b) A plot of the measured voltage over the diode while moving a charged PVC tube at different speeds. The dashed line in the middle separated the moving directions from left to right (left side of the dashed line) and from right to left (right side of the dashed line). The charge density of the PVC tube was 20 13.45 C·m^−2^ and vertical distance (*l*) was 0.5 cm. c) A plot of the maximum voltage versus the moving speed of the PVC tube, showing a linear relationship. d) Simulated electrostatic field of a charged circle at different values of *l*. e) Simulated potential at the red and blue points (1 µm in between, which is the thickness of the depletion layer) versus the position of the charge circle and f) The measured potential difference versus the position of a charged PVC tube. g) A plot of the potential difference versus 1/*l*. h) A schematic drawing shows the change of electrostatic field on a diode due to the in‐situ generated triboelectric charge by rubbing a dielectric material on another one. i) The potential at the red and blue points (0.001 mm in between) versus the distance between the diode and the dielectric material. j) The difference in potential of the two points versus the position of the slider (positive charged) in the simulation. k) The measured potential difference versus the position of the slider. Here we rub a piece of polyurethane (3 cm × 5 cm) on a polymethyl methacrylate plate. l) A plot of the potential difference versus 1/*l*. m) The Measured potential at different speeds of rubbing.

DiCes generate electricity by responding to time‐varying electrostatic fields. When a charged object moves near a diode junction, it modulates the electrostatic potential across the junction, inducing a voltage difference. This results in charge transport similar to an externally applied bias. **Figure** **1**a shows a diagram of a point charge moving parallel to a DiCe contains only one diode at a vertical distance of *l*. We used point charge in the development of the theoretical framework development because the distribution of its electrostatic field is well defined, which make it easy to explain the working mechanism. In experiment that described below, we used a charge tube to demonstrate the theoretical framework because it is easy to be operated and the results can explain the developed theoretical framework very well.

The point charge created a potential difference across the diode junction via electrostatic induction. The electrostatic potential (Φ) difference across the junction due to point charge (*q*) can be calculated by:

(1) $$\Delta \Phi =\Phi_{p}\;\left(r_{p},t\right)-\Phi_{n}\left(r_{n},t\right)=\frac{q}{4\pi \varepsilon }\left[\frac{1}{\left|r_{p}-r_{q}\left(t\right)\right|}-\frac{1}{\left|r_{n}-r_{q}\left(t\right)\right|}\right]$$

where *r_q_* (*t*)is the position of the moving charge at time *t*, *r_p_* and *r_n_* are the positions where the potential at the *p* and *n* side of a diode, ε is the permittivity of the surrounding medium. If the point charge is moving at a speed of *v*, the induced potential over the junction (*V_pn_* (*t*)can be given by:

(2) $$V_{pn}\;\left(t\right)=\frac{q}{4\pi \varepsilon }\;\left[\frac{1}{\sqrt{{\left(x-\frac{d}{2}-vt\right)}^{2}+l^{2}}}-\frac{1}{\sqrt{{\left(x+\frac{d}{2}-vt\right)}^{2}+l^{2}}}\right]$$

where *x* is the initial lateral position of the charge to the middle of *pn* junction, *d* is the width of the depletion layer (*d* = 1 µm), ε is the permittivity of air. If *V_pn_* (*t*) is the same as the forward bias direction, it will not over forward bias limit. If the diode is in a circuit, the current *I(t)* can be expressed by:

(3) $$I\left(t\right)=\left\{\begin{array}{c} C\frac{dV_{pn}}{dt},\;\;if\;0<V_{pn}<V_{f}\\ \frac{V_{f}}{R_{s}},\;\;if\;V_{pn}\geq V_{f}\\ M\left(V_{pn}\right)C\frac{dV_{pn}}{dt},\;\;\;if\;V_{pn}\leq -V_{br} \end{array}\right.$$

Where *C* is junction capacitance, *V_f_* is the forward bias threshold, *V_br_* is the breakdown voltage, *R_s_* is the resistance of the circuit, *M*(*V_pn_*) is the avalanche multiplication factor. For a DiCe contains *N_d_* serially connected diodes, the voltage can be expressed as

(4) $$V_{Dice}=\sum_{i=1}^{N_{d}}V_{pn}^{\left(i\right)}$$

and Equation (3) can be revised as:

(5) $$I_{DiCe}\left(t\right)=\left\{\begin{array}{c} \sum_{i=1}^{N_{d}}C\frac{dV_{pn}^{\left(i\right)}}{dt},\;\;if\;0<V_{pn}^{\left(i\right)}<V_{f}\\ N_{d}\frac{V_{f}}{R_{s}},\;\;if\;V_{pn}^{\left(i\right)}\geq \;V_{f}\\ \sum_{i=1}^{N_{d}}M\left(V_{pn}\right)C\frac{dV_{pn}^{\left(i\right)}}{dt},\;\;if\;V_{pn}^{\left(i\right)}\leq \;-V_{br} \end{array}\right.$$

## 2 Results and Discussion

### 2.1 Experimental Validation and the Performances of DiCes

According to Equation (2), the voltage across the diode is expected to have a nonlinear relationship with *v*. However, experimental results instead showed linear relationship (Figure 1b,c; Figure S1, Supporting Information), which may be due to the charged object being a polyvinyl chloride (PVC) tube rather than a point charge. The area between the voltage curve and the axis in Figure 1b represents the total inducted charge on the diode, which was found to be proportional to speed (Figure S2, Supporting Information). Equation (2) also predicts that voltage should exhibit a nonlinear relationship with 1/*l*, where a smaller *l* leads a higher voltage. Simulation results in Figure 1d,e show a simulated electrostatic field generated by a charged circle at different *l* values ranging from 1 to 5 cm, along with the voltage distribution relative to the position of the charge. The charge density was set at 13.45 C·m^−2^ based on the experimental results described below.

In the experimental setup, a charged PVC tube (ϕ  =  20 *mm*) with a charge density of 13.45 C·m^−2^ was moved above a diode. A plot of the measured potential versus positions (Figure 1f) exhibited a shape similar to the simulation, although the measured values were lower. It appears that the measured voltage was approximately 1/2000 of the simulated potential difference. Additionally, experimental results have confirmed that the potential difference was linearly correlated with 1/*l* (Figure 1g). (Figure S3, Supporting Information).

We also measured the voltage across other diodes under the same experimental conditions, in addition to the 1N4001 *pn*‐junction diode used in previous tests. Results indicated that the electric signals measured across different diodes were generally at the same level. However, for through‐hole *pn*‐junction diodes, the measured potential across the junction increased with increasing reverse breakdown voltage (Figures S4 and S5, Supporting Information).

Moving a charged tube above a diode, as described previously, is one of two methods for creating a changing electrostatic field. The other method involves generating an electrostatic field through in‐situ triboelectric charging (as shown in Figure 1h). A simulation illustrating the changes in the electrostatic field during the rubbing of one dielectric material against another is presented in Figure 1i. The surface charge density used in the simulation was set at 10.7 C·m⁻^2^, a value derived from experimental results.

Similar to the previous case, the distance (*l*) between the diode and the dielectric material played a crucial role in determining the intensity of the output electric signals, as confirmed by the simulation (Figure 1j). Interestingly, the ratio between the measured voltage and the simulated voltage was again approximately 1/2000, identical to the previous case. Experimental results also confirmed the significance of the distance (Figure 1k), demonstrating a linear relationship between potential intensity and 1/*l* (Figure 1l), as well as between potential intensity and rubbing speed (Figure 1m; Figure S6, Supporting Information). Interestingly, unlike the case of a moving PVC tube, the electric signals differed between different types of diodes (Figure S7, Supporting Information). This variation may be attributed to differences in the distribution of the electric field.

### 2.2 Energy Harvesting Using Diode Cell (DiCe)

**Figure 2:**
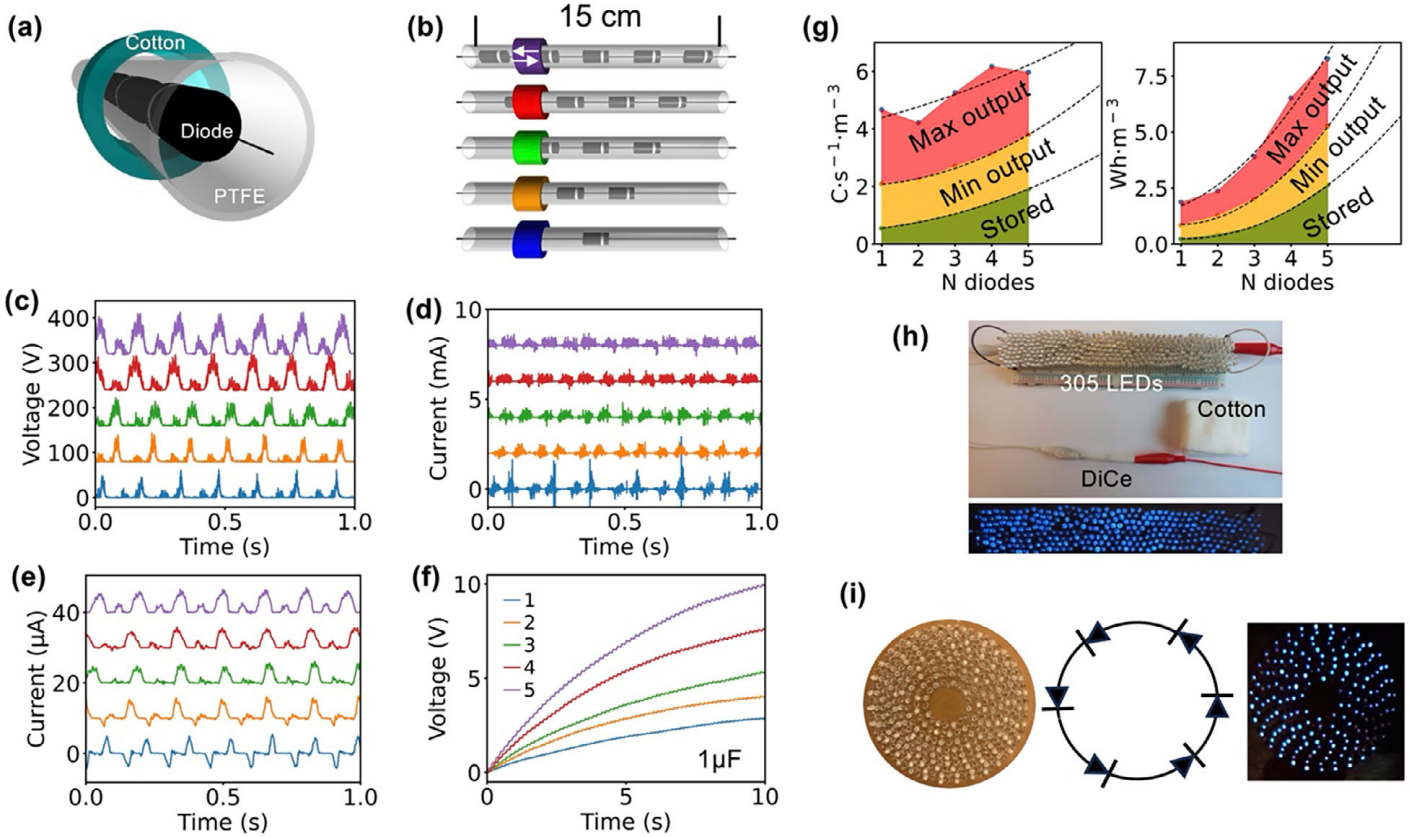
Performance of DiCes. a) A schematic drawing of the DiCe, where diodes are serially connected and put inside a PTFE tube. b) Five DiCes constructed with 1 to 5 diodes. The coloured cylinder represents a cotton wrapped on the PTFE tube, and the colours correlated to the colours in c–f). c) Open circuit voltage measured on the DiCes. The order of signal corresponds to the order in b). d) Short circuit current measured on the DiCes. e) Short circuit current measured on the DiCes after filtering the high frequency signals. f) Voltage measured on a 1 µF capacitor charged with the DiCes. g) Charge generation and Energy output of the DiCe per second at a volume of 1 cubic meter. h) A photograph of the circuit for powering 305 LEDs with a single‐diode DiCe. i) A DiCe consists of 9 LED loops with close circuits, the circuit of the loops, and LEDs lit up by rubbing a piece of cotton on the PTFE film that attached to the back of the DiCe.

The simulations and experimental results presented above confirm that a diode can generate electricity in a dynamically changing electrostatic field. Furthermore, in both previously discussed cases, the diode remained stationary. To better understand the differences in energy harvesting between these scenarios, a diode inside a polytetrafluoroethylene (PTFE) tube was fabricated, serving as a DiCe (**Figure** **2**a). A piece of cotton was used to rub the PTFE tube, creating a variable electrostatic field through triboelectrification. The cotton was used because of two reasons: 1) Cotton is a relatively triboelectric positive material which can have a higher triboelectric effect with the PTFE. 2) the DiCes that made in our experiment are relatively with big area, which make them not easy to be made very flat, especially for the 3D printed frames. Using cotton as the rubbing material reduced the influence of the geometry of the DiCes.

To study the performance of a DiCe assembled with multiple diodes, we fabricated four additional DiCes containing one to five diodes (Figure 2b). Experimental results revealed that the open‐circuit voltages (Figure 2c) of the DiCes depended on the number of diodes, which was consistent with Equation (4). As the rubbing motion occurred back and forth at a frequency of 7 Hz, voltage signals appeared as alternating strong and weak peaks. Both the intensity and width of these peaks correlated with the number of diodes in the DiCe, revealing a non‐linear relationship for intensity and a linear relationship for signal width (Figures S8 and S9, Supporting Information). The peaks observed in DiCes with multiple diodes represented an overlay of signals from individual diodes, explaining the linear relationship between signal width and diode count (Figure S10, Supporting Information).

The maximum short‐circuit current of the DiCes reached milliamperes levels (Figure 2d). The current signals contained peaks in both high‐frequency and low‐frequency domains. High‐frequency signals were attributed to electrostatic discharge during contact electrification, occurring consistently. Conversely, low‐frequency signals, which remained after high‐frequency components were filtered out (Figure 2e), were associated with electrostatic induction and exhibited greater stability. Notably, DiCes with one or two diodes exhibited AC current signals, while those with three or more diodes displayed DC signals. This behavior may be linked to characteristic changes in DiCes, as illustrated in Figure S11 (Supporting Information), which depicts source‐drain characterization. These results suggest that increasing the number of diodes and extending the PTFE tube enables continuous DC current generation (Figure S12, Supporting Information). Alternatively, a ring‐structured DiCe could be designed to generate continuous DC current for powering electronics (Video S1, Supporting Information).

The energy output of the five DiCes was evaluated by charging a 1 µF capacitor for 10 s (Figure 2f). Results indicated that the stored energy was proportional to the number of diodes in the DiCes. For a DiCe with five diodes, the charge stored in a 1 µF capacitor after 10 seconds reached over 1.8 C·s⁻¹·m⁻^3^, equivalent to 2.5 Wh·m⁻^3^ (Figure 2g). The DiCe was 15 cm long and, in principle, could accommodate at least 10 diodes, suggesting that the energy output could exceed 10 Wh·m⁻^3^, based on the fitted curve for capacitor‐stored energy. The maximum output power of a single‐diode DiCe reached 680 mW, which corresponded to 362 W·m⁻^2^ when accounting for the surface area of the PTFE tube (radius = 2 mm), or 362 kW·m⁻^3^ when considering the volume. Filtering out the high‐frequency signal reduced the power to 10.5 mW, corresponding to 5.6 W·m⁻^2^ and 5.6 kW·m⁻^3^, respectively. The high‐power output capability was demonstrated by powering 305 LEDs with a single‐diode DiCe (Figure 2h). Voltage and current measurement results, along with a demonstration video, are provided in Supplementary Figure S13 and Video S2 (Supporting Information). Taking advantage of the current modulation characteristic of diodes, a DiCe with a closed‐loop LED configuration (Figure 2i) was successfully illuminated by rubbing the backside of a PTFE film attached to the DiCe with a piece of cotton (Video S3, Supporting Information). This represents a novel strategy for lighting LEDs without a conventional power source.

**Figure 3:**
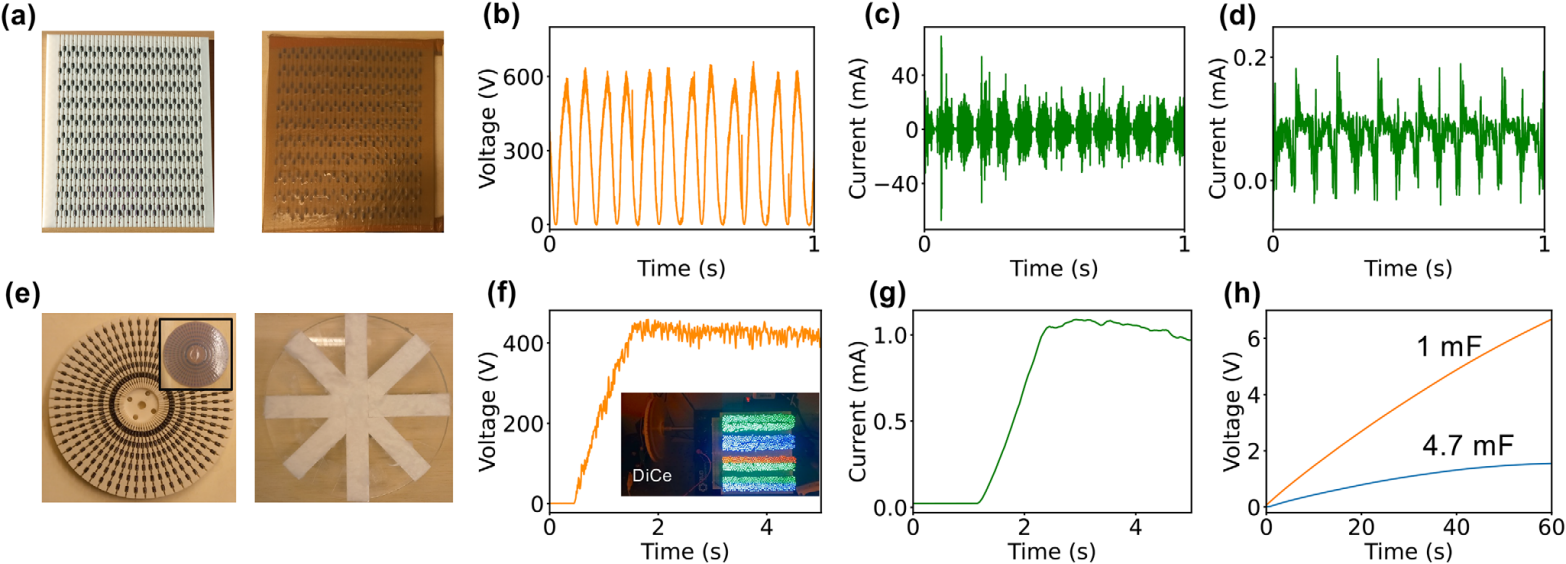
Energy harvesting with DiCes. a) Photographs DiCe‐I with 10 × 34 diodes without and with a cover of PTFE film. b) Open circuit voltage of the DiCe by rubbing a 3 cm × 18 cm cotton on it. c) Short circuit current of the DiCe before and d) after filtering away the signals at high frequency region. e) Photographs DiCe‐II with 6 × 36 diodes without and with a cover of PTFE film (insert), and the counter cotton plate used to rotate on the DiCe. f) Open circuit voltage of the DiCe measured at a rotation speed of 1152 rpm. The insert shows a photograph of more than 1480 LEDs lit up by the DiCe. g) Short circuit current of the DiCe. h) Charge 1 and 4.7 mF capacitors with the DiCe.

The above results confirm that the power and energy output of a DiCe strongly depend on the number of diodes. To achieve higher outputs, two DiCes with different constructions (**Figure** **3**a,e) were developed, containing 340 (DiCe‐I) and 360 (DiCe‐II) diodes, respectively. Sliding a cotton strip over DiCe‐I generated voltage and current outputs (Figure 3b), reaching up to 709 V, 68.6 mA in the high‐frequency domain, and 0.17 mA in the low‐frequency domain (Figure 3c,d). DiCe‐II was designed as a disk and operated similarly to a rotating TENG. However, unlike a rotating TENG, DiCe‐II generated DC voltage directly (Figure 3f) and produced a maximum current of 1.08 mA (Figure 3g), resulting in a power density of 26.5 W·m⁻^2^ at a rotation speed of 1152 rpm. With this power density, DiCe‐II was able to charge a 4.7 mF capacitor to 1.5 V within 60 seconds (Figure 3h). As a DC power source, the DiCe‐II can directly power 1480 LEDs (Figure 3f) without flashing (Supplementary Video S4, Supporting Information). Our results indicated that after 50 000 cycles, the output current of the DiCe remained up to 95% of the original value (Figure S14, Supporting Information). The decrease may come from the shape change of the cotton strips because the fibers could re‐arrange the alignment during the operation of the system. The lowest rotation speed that was found to generate detectable electrical signal was 30 rpm.

### 2.3 Applications of DiCes

#### 2.3.1 Powering of Implanted Electronics

**Figure 4:**
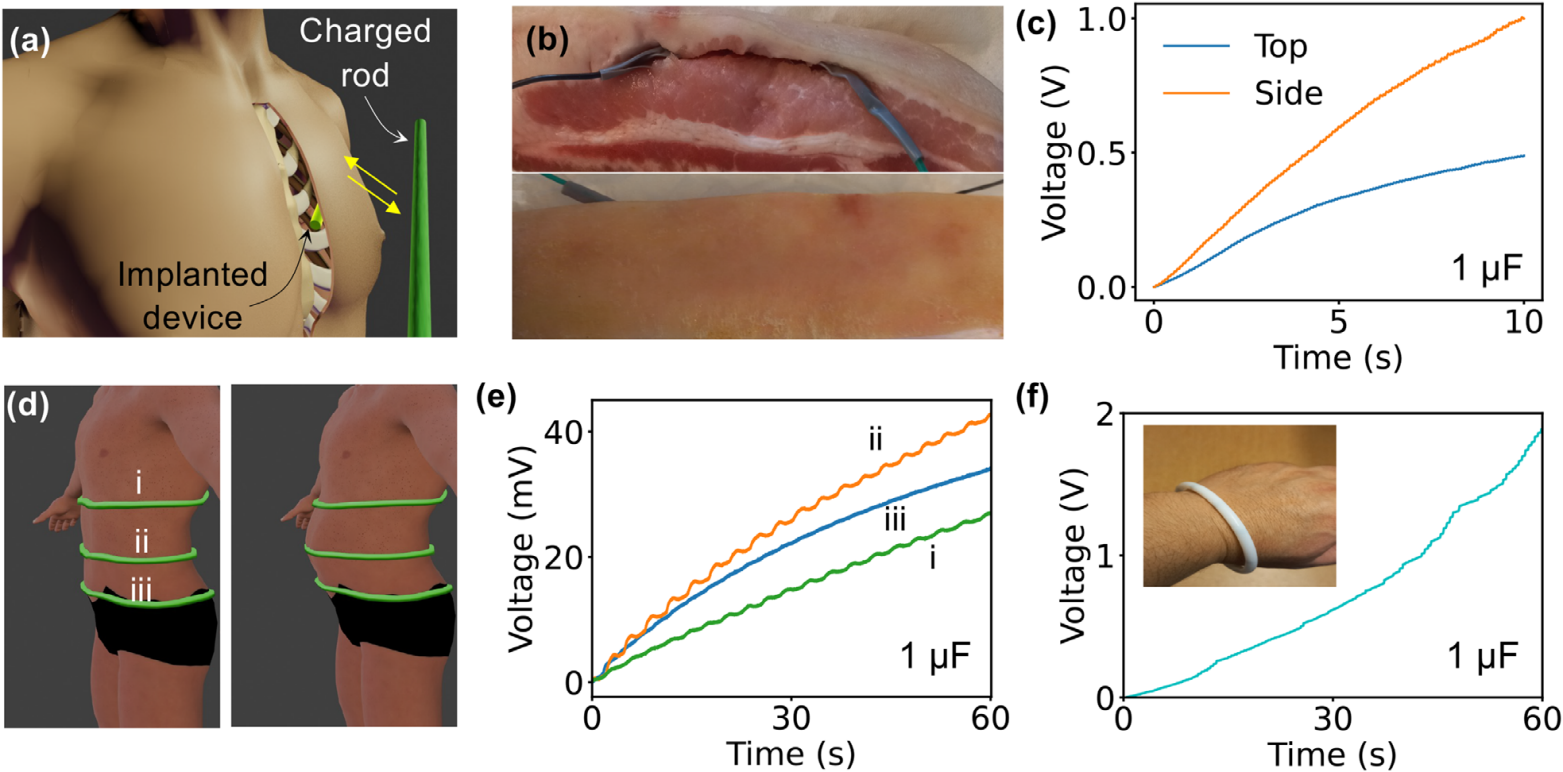
Powering of implanted electronics and applications in wearable electronics. a) An image shows the strategy of moving a charged rod at the place where an implanted device was placed. Image created with Blender 3D and Z‐anatomy.^[^ ^20^ ^]^ b) A photograph shows a single‐diode DiCe implanted in a piece of pork. The DiCe was connected to a 1 µF capacitor to store the generated charges. c) Charging the capacitor by moving a charged PVC tube above (skin) and to the side of the pork. d) A 3D model (created with Blender 3D and MB‐Lab^[^ ^21^ ^]^) shows the placing of a belt like DiCe at three places on the belly to harvest energy from respiration. e) Charge a 1 µF capacitor with the belt placed at different places for 60 s. f) A DiCe bracelet is worn at the wrist for harvesting energy from walking. The energy output was measured by charging a 1 µF capacitor while walking for 60 s. The human body model was created using Blender (version 4.0.2) and MB_Lab (Version 1.7.8).

We have demonstrated that a DiCe can harvest energy from a changing electrostatic field at a distance. This capability can be used to power implanted electronics (**Figure** **4**a). For example, a charged rod that moves near the body where a device is implanted could charge capacitors to power the device. To test this concept, we conducted a simple experiment by placing a single‐diode DiCe inside a piece of pork (Figure 4b) and moving a charged PVC tube above and beside it. A 1 µF capacitor was connected to the diode via a bridge rectifier to store the generated charge. Figure 4c shows two charging curves corresponding to the movement of the PVC tube above and beside the pork sample. In addition to using a charged tube, another option is to generate charge on‐site by rubbing two dielectric materials above the implantation site. Compared to wireless charging strategies^[^ ^22^, ^23^, ^24^, ^25^ ^]^ for implanted devices, our approach is simpler, more cost‐effective, and offers greater accessibility. The DiCe that we study here can be used as power source for implants. Practically, it will not work solely and will not be directly contacted with tissue. Instead, it could be encapsulated in bio‐compatible materials.

#### 2.3.2 Wearable Energy Harvesting Devices

A person typically consumes 2000 kcal of energy daily, with ≈24% to 32% allocated to physical movement, equating to an average of 2.34 MJ. Although efforts have been made to harvest energy from body motions using different technologies,^[^ ^13^, ^26^, ^27^, ^28^, ^29^ ^]^ challenges related to efficiency,^[^ ^30^ ^]^ simplicity,^[^ ^31^ ^]^ and comfort^[^ ^32^ ^]^ remain. One major challenge is that the physical movements vary significantly in time and intensity, making it hard to efficiently convert the mechanical energy to electricity. Devices such as TENGs and piezoelectric nanogenerators offer convenient ways to convert mechanical energy from human body motions into electricity. However, these devices require specific orientation during installation to maximize output. In contrast, a DiCe has fewer installation requirements as it can produce electricity by utilizing movement‐induced changes in the body's electrostatic potential.^[^ ^33^, ^34^, ^35^ ^]^

To demonstrate this, we designed a DiCe in the form of a belt and wore it on the waist, belly, and chest to harvest energy from respiration (Figure 4d,e). This belt generated ≈40 nC over 60 s, which corresponds to 1.15 µJ per day. The output could be significantly increase by including more diodes than the experiment (30 diodes) in DiCe, as we have proved above that the output is dependent on the number of diodes. Although the energy output is relatively low, the belt could be repurposed as a respiratory sensor,^[^ ^36^, ^37^, ^38^, ^39^ ^]^ allowing for the extraction and analysis of intensity and frequency data. In another experiment, we created a DiCe in the form of a bracelet to harvest energy while a person was walking. Over 60 s at a frequency of two steps per second, a 1 µF capacitor was charged to ≈1.8 V (Figure 4f), demonstrating the feasibility of the strategy.

#### 2.3.3 Spontaneous Energy Harvesting from Cars and Roads

DiCes are a types of stationary energy harvesting device that requires no displacement of components (unlike TENGs^[^ ^40^ ^]^) and does not require mechanical work to function. This characteristic makes them easy to integrate with other objects. DiCes could be mounted inside car tires and under a road to spontaneously harvest energy while a car is running on them. Both simulation and experimental results have demonstrated the feasibility of this energy‐harvesting method. Under ideal conditions, an electric car can recover ≈10% of the energy lost on rolling resistance. Detailed simulations and results are provided in Supplementary: Energy recycling on tires and road.

## 3 Discussions

A debate on how similar the DiCes are to rectenna arose during our study. Rectennas^[^ ^41^, ^42^, ^43^ ^]^ use the polarization of two antenna electrodes by the electric field of the incoming radio wave. Such a mechanism requires the alignment of the antenna to the electric field.^[^ ^44^ ^]^ For DiCes, however, such alignment is unnecessary, as experiments have proven that the electrostatic field can be either parallel or perpendicular to the diode electrodes. However, compared to the rectenna, the DiCe has a shorter working distance because the intensity of the electrostatic field decreases rapidly with distance.

Environmental factors such as temperature and humidity were found to have limited influence on DiCe devices operated through direct rubbing of cotton against PTFE. However, for DiCe devices operating at a distance—such as those utilizing a charged PVC tube—humidity was observed to have a significant impact. We conducted two experiments at relative humidities of 30% and 80%, respectively. The results showed that at 80% humidity, the surface charge density decreased by more than 50% after 10 min. In contrast, at 30% humidity, the decrease was only ≈10% over the same period.

Compared to TENGs and piezoelectric nanogenerators (PENGs), DiCes are stationary devices that require no mechanical movement. Additionally, DiCes have unique characteristics (Supplementary: Unique Features of the DiCes), such as free motion on surfaces and in space, which generates dynamically changing electrostatic fields in any direction. They also exhibit a volume effect, allowing multiple DiCes to be stacked to enhance energy harvesting, as well as the ability to harvest energy at a distance. A table that compares the performance of DiCe with recently developed TENGs and PENGs is given in the supporting information.

Moreover, both theoretical analysis and experimental results have demonstrated that DiCe output is highly dependent on the number of diodes. Commercial diodes are encapsulated in plastic, which has a significantly larger volume than the diode itself. If the volume were reduced, the diode density per unit area could be increased multiple times, leading to significantly higher energy output from the DiCes. Moreover, large‐scale DiCe can be simply made by increasing the number of diodes, allowing higher energy output. Our experimental results have shown that the output of a DiCe is proportional to the number of diodes that used. Therefore, it is easy to gain a high output by enlarge the size of DiCe to include more diodes.

The working mechanisms of the DiCe allow it to be hybridized with TENGs because they generate dynamically changing electrostatic field. Such change electrostatic field could be adopted by the DiCe to generate electricity. However, specific design of the hybridized device needs to be made because the large area electrode that used in TENG may block the distribution of the electrostatic field.

## 4 Conclusion

In summary, we have developed Diode Cells (DiCes) as a method for harvesting energy from distributed mechanical motions, addressing the growing demand for alternative power sources. The working mechanisms of DiCes have been proposed, simulated, and experimentally verified. The unique features of DiCes were also identified, such as their operational versatility in free‐motion scenarios and the volume effect, which differentiates them from other energy‐harvesting technologies. DiCes demonstrate diverse applications, from powering implanted electronics to harvesting energy from vehicles and roads, showcasing their adaptability and effectiveness in a range of environments. While experimental results validate the feasibility and effectiveness of DiCes, further research is needed to optimize their performance, improve energy storage efficiency, and confirm real‐world applicability. Overall, DiCes represent a promising solution for sustainable energy generation, with the potential to contribute significantly to renewable energy advancements. They offer an innovative approach to powering sensors, small electronics, and infrastructure components,

## 5 Experimental Section

### Simulation

All simulation was done by using COMSOL Multiphysics version 6.2. To simulate the voltage difference across a diode, two metal wires with 0.001 mm in between were placed in the simulation model. The distance was used because the thickness of the depletion layer of a *pn* diode is usually 0.001 mm.

To simulate the moving of a PVC tube above a diode, a circle with a diameter of 2 cm which is the same as the PVC tube used in our experiments was used. The surface charge density was set to the value that was measured on the PVC tube after rubbing it with a piece of cat fur (borrowed from the student lab). The surface charge was measured using a Faraday cup (Electro‐Tech Systems) that was connected to an electrometer (Keithley 6514). The vertical distances between the PVC tube and the diode were set as 1, 2, 3, 4, and 5 cm.

To simulate the scenario that a diode is placed under a piece of PTFE plate while a piece of cotton is rubbing on the PTFE, the surface charge density was also taken from the experimental results. The distance between the diode and the PTFE plate was set as 1, 2, 3, and 4 cm. To simulate the rolling of a tire on a road, the surface charge density was set at 1 nC·m^−2^ for the road and −1 nC·m^−2^ for the tire. The rolling speed is not included in the simulation.

### Materials and Measurement

All diodes and LEDs were purchased from Elfa. PTFE was purchased from High‐Tech Flon. PVC tube was purchased from McMaster‐Carr. Cat fur that was used for charging the PVC tube was borrowed from the student lab.

Electric measurements were done with a PXI 4071 digital multimeter (National Instruments) at a sampling ratio of 200 000 s^−1^ for open circuit voltage and short circuit current measurement and 1000 s^−1^ for capacitor charging experiments. The multi‐channel electric measurement for the experiment described in Figure 3i was done by using a MCC DAQ device (MCC USB‐1604HS‐2AO). The measurements for DiCe‐II were done using a DMM7510 multimeter (Keithley) for voltage measurement and a 6514 electrometer (Keithley) for current measurement. The rotation of the disks was controlled by using a rotation system (Beijing Naneng Instrument Technology Co. Ltd.). The diameter of the DiCe and rotation disk was 16 cm. The size of cotton strips was 2 cm × 7.5 cm.

The frames for DiCes in Figure 3 were printed on a Wanhao Duplicator i3 plus 3D printer using PLA filament.

### Surface Charge Measurement

To do surface charge measurement, a charged PVC tube and PTFE were put inside the Faraday's cup that was connected to a Keithley 6514 electrometer. The charge density was obtained by dividing the total charge with the rubbed area of the PVC tube.

## Conflict of Interest

R. Y. Zhang has submitted a US provisional patent application (application number: 63/709448).

## Supporting information



Supporting Information

Supplemental Video 1

Supplemental Video 2

Supplemental Video 3

Supplemental Video 4

Supplemental Video 5

## Data Availability

The data that support the findings of this study are available from the corresponding author upon reasonable request.

## References

[1] C. Wu , A. C. Wang , W. Ding , H. Guo , Z. L. Wang , Adv. Energy Mater. 2019, 9, 1802906.

[2] M. Gorlatova , J. Sarik , G. Grebla , M. Cong , I. Kymissis , G. Zussman , IEEE J. Sel. Areas Commun. 2015, 33, 1624.

[3] S. Choo , F. Ejaz , H. Ju , F. Kim , J. Lee , S. E. Yang , G. Kim , H. Kim , S. Jo , S. Baek , S. Cho , K. Kim , J. Y. Kim , S. Ahn , H. G. Chae , B. Kwon , J. S. Son , Nat. Commun. 2021, 12, 3550. 34112808 10.1038/s41467-021-23944-w PMC8192747

[4] Y. Zhang , T. Yang , K. Shang , F. Guo , Y. Shang , S. Chang , L. Cui , X. Lu , Z. Jiang , J. Zhou , C. Fu , Q. C. He , Nat. Commun. 2022, 13, 3484. 35710907 10.1038/s41467-022-31067-z PMC9203740

[5] M. A. Weimer , T. S. Paing , R. A. Zane , in 2006 37th IEEE Power Electronics Specialists Conference , IEEE, 2006, pp. 1–5.

[6] H. C. Ates , P. Q. Nguyen , L. Gonzalez‐Macia , E. Morales‐Narváez , F. Güder , J. J. Collins , C. Dincer , Nat. Rev. Mater. 2022, 7, 887. 35910814 10.1038/s41578-022-00460-x PMC9306444

[7] H. Sheng , X. Zhang , J. Liang , M. Shao , E. Xie , C. Yu , W. Lan , Adv. Healthcare Mater. 2021, 10, 2100199. 10.1002/adhm.202100199 33930254

[8] Q. Zheng , Q. Tang , Z. L. Wang , Z. Li , Nat. Rev. Cardiol. 2021, 18, 7. 32895536 10.1038/s41569-020-0426-4

[9] R. Liu , Z. L. Wang , K. Fukuda , T. Someya , Nat. Rev. Mater. 2022, 7, 870.

[10] H. Wu , Y. Huang , F. Xu , Y. Duan , Z. Yin , Adv. Mater. 2016, 28, 9881. 27677428 10.1002/adma.201602251

[11] H.‐X. Zou , L.‐C. Zhao , Q.‐H. Gao , L. Zuo , F.‐R. Liu , T. Tan , K.‐X. Wei , W.‐M. Zhang , Appl. Energy 2019, 255, 113871.

[12] F.‐R. Fan , Z.‐Q. Tian , Z. L. Wang , Nano Energy 2012, 1, 328.

[13] R. Zhang , J. Örtegren , M. Hummelgård , M. Olsen , H. Andersson , H. Olin , Nano Energy 2018, 45, 298.

[14] X. Cao , H. Xiang , P. Ma , Y. Jie , Y. Zhang , H. Guo , N. Zheng , Z. L. Wang , Nano Energy 2022, 100, 107409.

[15] H. Xiang , L. Peng , Q. Yang , Z. L. Wang , X. Cao , Sci. Adv. 2024, 10, 2291. 10.1126/sciadv.ads2291 PMC11606449 39612344

[16] T. Huang , C. Wang , H. Yu , H. Wang , Q. Zhang , M. Zhu , Nano Energy 2014, 14, 226.

[17] W. Wang , J. Zhang , Y. Zhang , F. Chen , H. Wang , M. Wu , H. Li , Q. Zhu , H. Zheng , R. Zhang , Appl. Phys. Lett. 2020, 116, 023901.

[18] E. Su , S. Xu , Z. Wang , Z. Xu , S. Pan , Z. L. Wang , L. N. Y. Cao , Mater. Sci. Eng.: R: Rep. 2025, 164, 100953.

[19] J. P. Das , S. Nardekar , V. Ravichandran , S.‐J. Kim , J. P. Das , S. S. Nardekar , V. Ravichandran , S.‐J. Kim , Small 2024, 20, 2405792. 10.1002/smll.202405792 39221685

[20] Blender Online Community , http://www.blender.org 2018.

[21] MB‐Lab Development Team , MB‐Lab: Advanced 3D humanoid creation tool for Blender (Version 1.7.8) [Software] 2024, https://github.com/animate1978/MB‐Lab.

[22] D. Nikolayev , M. Zhadobov , P. Karban , R. Sauleau , Phys. Rev. Appl. 2018, 9, 024033.

[23] K. Agarwal , R. Jegadeesan , Y.‐X. Guo , N. V. Thakor , IEEE Rev. Biomed. Eng. 2017, 10, 136. 28328511 10.1109/RBME.2017.2683520

[24] C. Y. Kim , M. J. Ku , R. Qazi , H. J. Nam , J. W. Park , K. S. Nam , S. Oh , I. Kang , J.‐H. Jang , W. Y. Kim , J.‐H. Kim , J.‐W. Jeong , Nat. Commun. 2021, 12, 535. 33483493 10.1038/s41467-020-20803-y PMC7822865

[25] R. Bashirullah , IEEE Microw. Mag. 2010, 11, S14.

[26] M. Cai , Z. Yang , J. Cao , W.‐H. Liao , Energy Technol. 2020, 8, 2000533.

[27] R. Riemer , A. Shapiro , J. Neuroeng. Rehabil. 2011, 8, 22. 21521509 10.1186/1743-0003-8-22 PMC3098156

[28] R. Zhang , M. Hummelgård , J. Örtegren , M. Olsen , H. Andersson , Y. Yang , H. Zheng , H. Olin , Nano Energy 2021, 86, 106041.

[29] R. Zhang , M. Hummelgård , J. Örtegren , M. Olsen , H. Andersson , Y. Yang , H. Olin , ACS Appl. Energy Mater. 2018, 1, 2955.

[30] S. Khalid , I. Raouf , A. Khan , N. Kim , H. S. Kim , Int. J. Precis. Eng. Manufacturing‐Green Technol. 2019, 6, 821.

[31] Y. Zou , L. Bo , Z. Li , Fundam. Res. 2021, 1, 364.

[32] B. Yang , Y. Xiong , K. Ma , S. Liu , X. Tao , EcoMat 2020, 2, 12054.

[33] T. Mizuno , K. Takashima , A. Mizuno , J. Electrostat. 2013, 71, 524.

[34] T. Ficker , J. Electrostat. 2006, 64, 10.

[35] R. Zhang , M. Hummelgård , J. Örtegren , Y. Yang , H. Andersson , E. Balliu , N. Blomquist , M. Engholm , M. Olsen , Z. L. Wang , H. Olin , Nano Energy 2019, 63, 103842.

[36] H. Zhang , J. Zhang , Z. Hu , L. Quan , L. Shi , J. Chen , W. Xuan , Z. Zhang , S. Dong , J. Luo , Nano Energy 2019, 59, 75.

[37] S. Wang , H. Tai , B. Liu , Z. Duan , Z. Yuan , H. Pan , Y. Su , G. Xie , X. Du , Y. Jiang , Nano Energy 2019, 58, 312.

[38] M. Wang , J. Zhang , Y. Tang , J. Li , B. Zhang , E. Liang , Y. Mao , X. Wang , ACS Nano 2018, 12, 6156. 29847095 10.1021/acsnano.8b02562 PMC6279609

[39] Y. Su , G. Chen , C. Chen , Q. Gong , G. Xie , M. Yao , H. Tai , Y. Jiang , J. Chen , Adv. Mater. 2021, 33, 2101262. 10.1002/adma.202101262 34240473

[40] X. Cao , M. Zhang , J. Huang , T. Jiang , J. Zou , N. Wang , Z. L. Wang , Adv. Mater. 2018, 30, 1704077. 10.1002/adma.201704077 29315850

[41] M. Wagih , A. S. Weddell , S. Beeby , IEEE Antennas Propag. Mag. 2020, 62, 95.

[42] C. Song , Y. Huang , J. Zhou , J. Zhang , S. Yuan , P. Carter , IEEE Trans. Antennas. Propag. 2015, 63, 3486.

[43] J. A. Hagerty , F. B. Helmbrecht , W. H. McCalpin , R. Zane , Z. B. Popovic , IEEE Trans. Microw Theory Tech. 2004, 52, 1014.

[44] S. D. Assimonis , V. Fusco , A. Georgiadis , T. Samaras , Sci. Rep. 2018, 8, 15038. 30301980 10.1038/s41598-018-33388-w PMC6177435

