# Supplementary material for: Harnessing Dynamic Electrostatic Fields for Energy Generation with Diode Cells
Source: Adv Sci (Weinh). 2025 May 14;12(28):e05476. doi: 10.1002/advs.202505476 (PMC12302571; doi:10.1002/advs.202505476)
Supplement: Supplementary file 1 — Supporting Information [file ADVS-12-e05476-s006.pdf]

ADVANCED SCIENCE
Open Access

Supporting Information

for *Adv. Sci.*, DOI 10.1002/advs.202505476

Harnessing Dynamic Electrostatic Fields for Energy Generation with Diode Cells

Renyun Zhang, Magnus Hummelgård, Ye Xu, Martin Olsen, Jonas Örtegren, Göran Thungström, Henrik Andersson and Zhong Lin Wang*

Supporting information

Harnessing Dynamic Electrostatic Fields for Energy Generation with Diode Cells

Renyun Zhang, Magnus Hummelgård, Ye Xu, Martin Olsen, Jonas Örtegren, Göran Thungström, Henrik Andersson, Zhong Lin Wang

FIGURE S1 2
FIGURE S2 3
FIGURE S4 5
FIGURE S5 6
FIGURE S6 7
FIGURE S7 8
FIGURE S8 9
FIGURE S9 10
FIGURE S10 11
FIGURE S11 12
FIGURE S12 13
FIGURE S13 14
FIGURE S14. 15
ENERGY RECYCLING ON TIRES AND ROAD 16
ENERGY RECYCLING ON TIRES 16
FIGURE S15 17
TABLE S1 18
FIGURE S16 19
ENERGY RECYCLING ON ROAD 20
UNIQUE FEATURES OF THE DICES 22
FIGURE S17 23
FIGURE S18 25
FIGURE S19 26
FIGURE S20 27
FIGURE S21 28
TABLE S2. 29

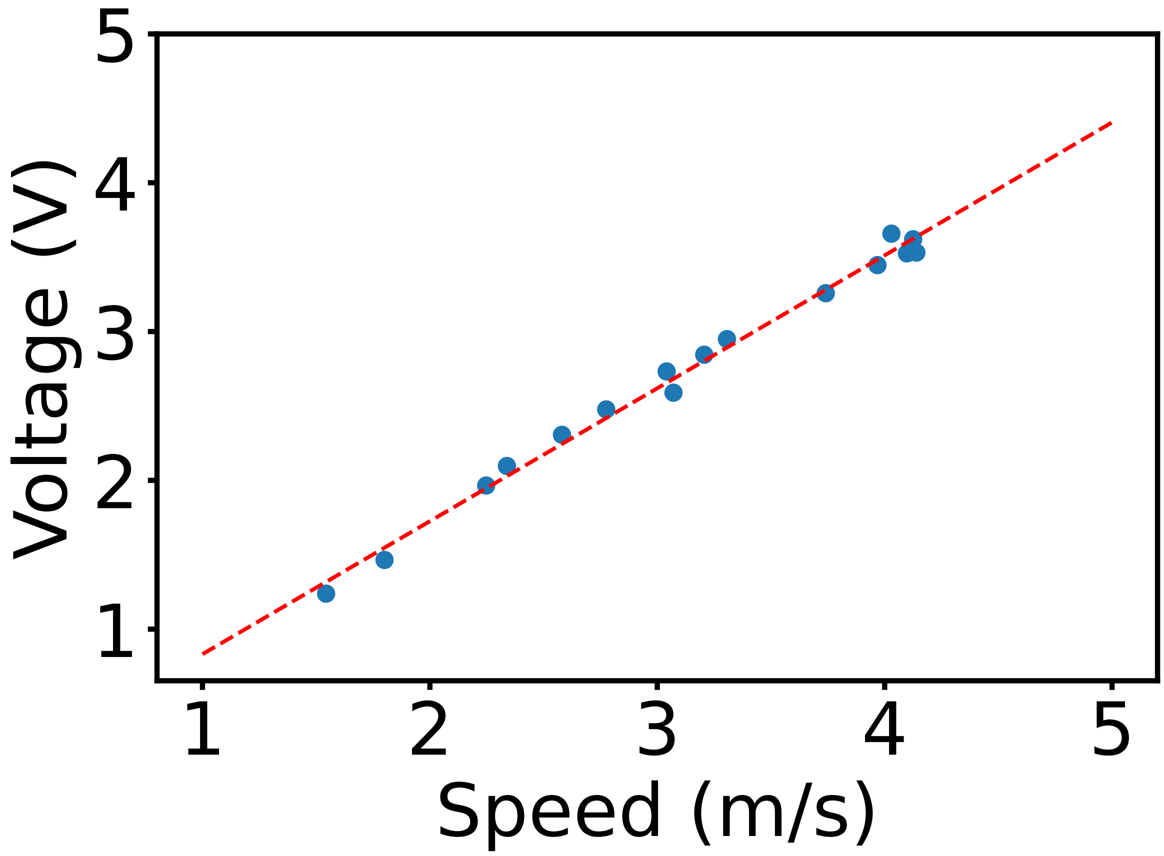

Figure S1. Measured voltage versus the moving speed of the charged PVC tube above a diode, showing a linear relationship.

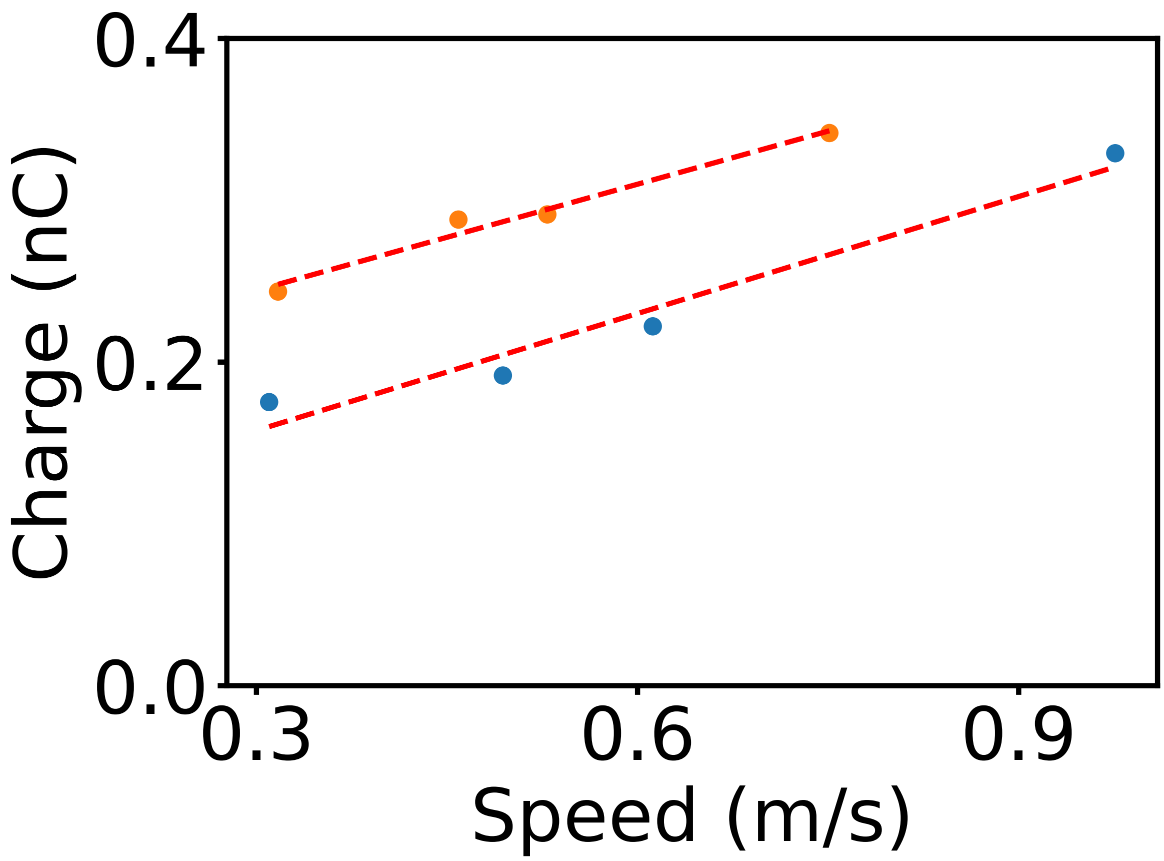

Figure S2. A plot of the induction charge on the diode vs the moving speed of the PVC tube.

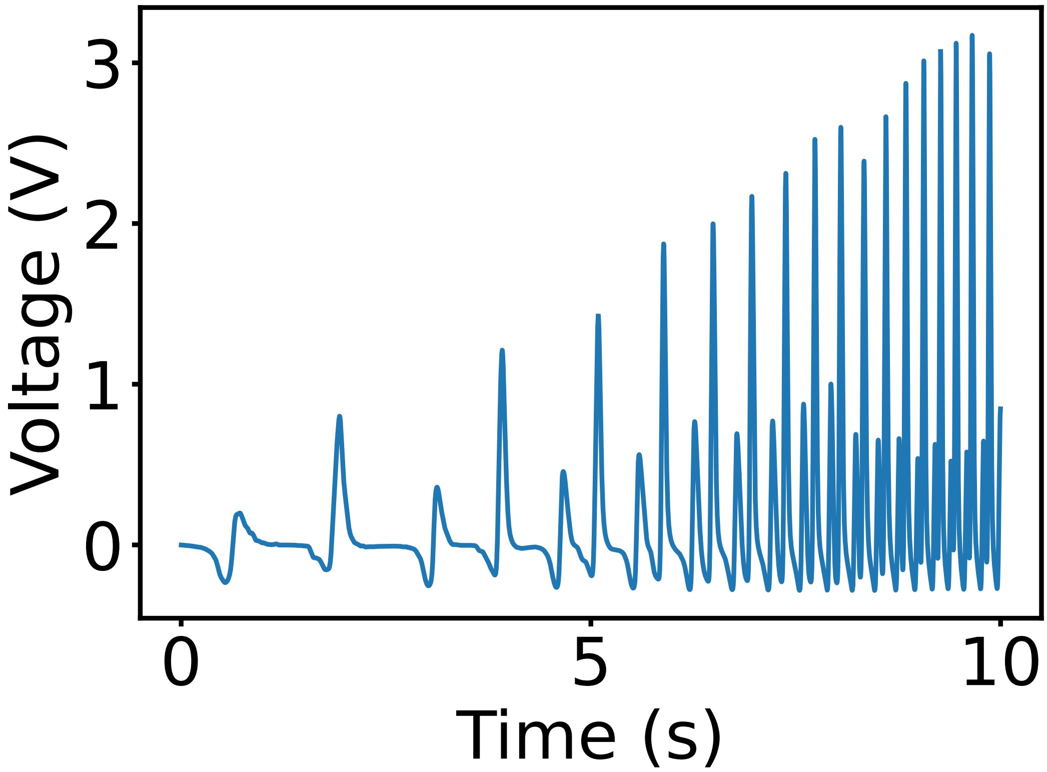

Figure S3. Measured voltage on the diode at different moving speed of a charged PVC tube.

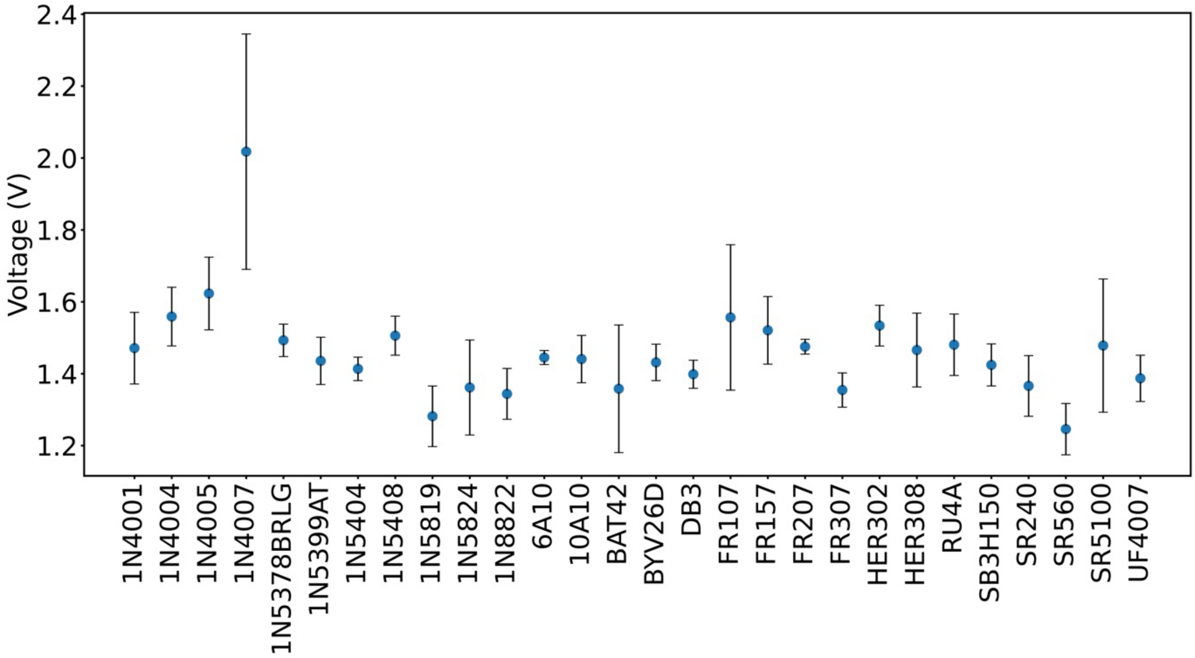

Figure S4. Voltage measured on different types of diodes while moving a charged PVC tube above the diodes.

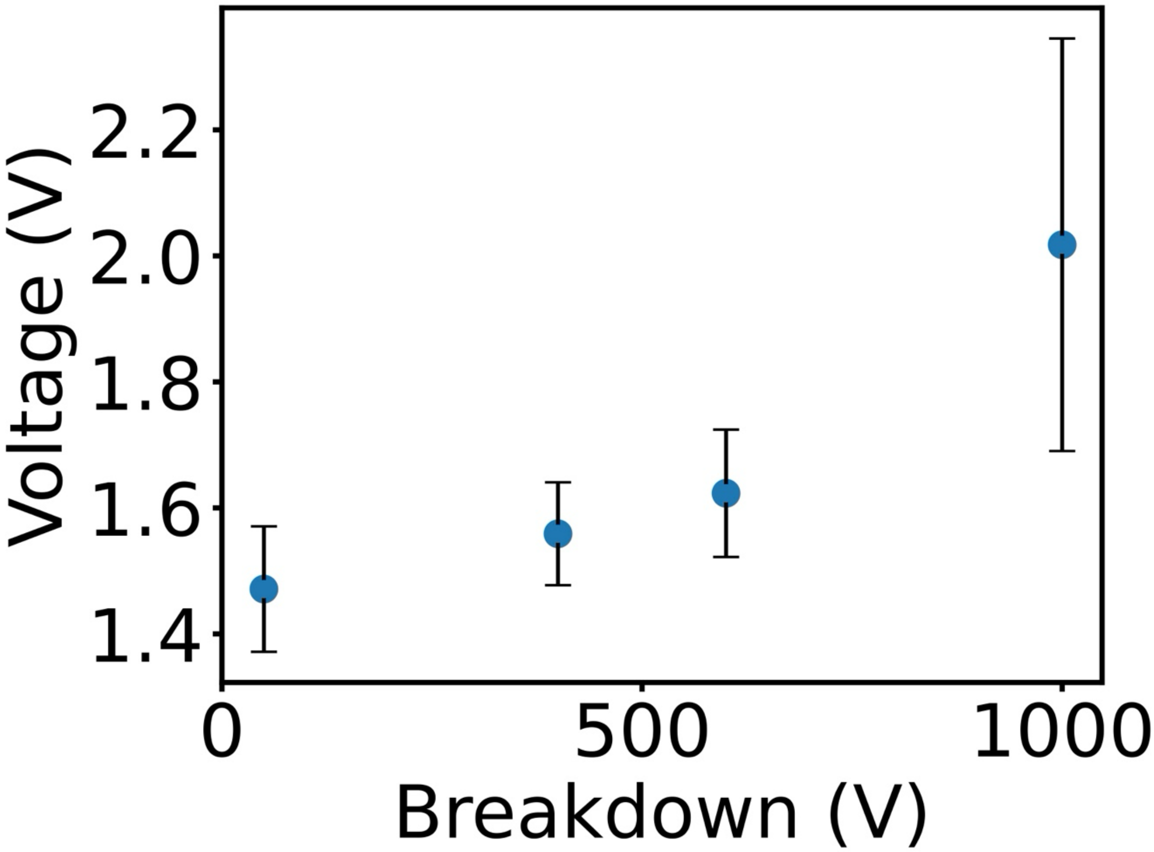

Figure S5. Voltage measured on through-hole *pn* junction diodes while moving a charged PVC tube above the diodes. From left to right: 1N14001, 1N14004, 1N14005, 1N14007.

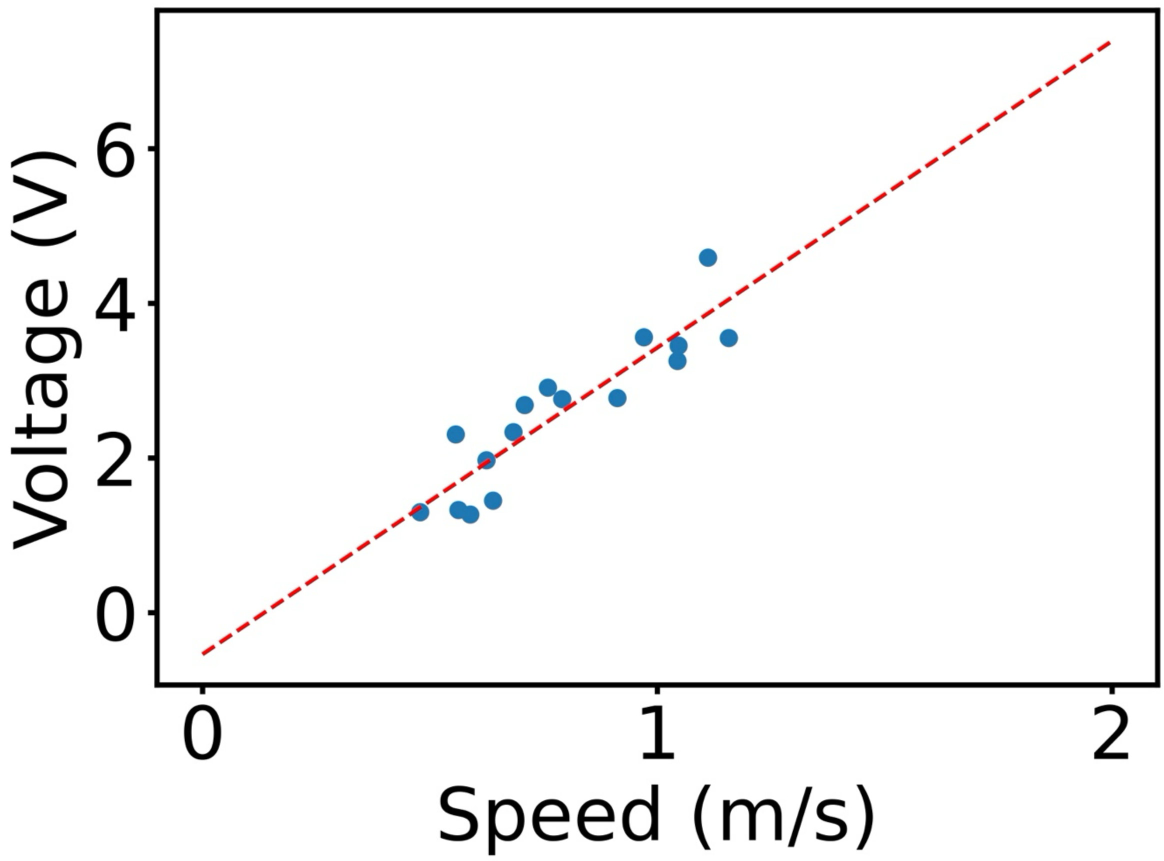

Figure S6. Measured voltage versus the moving speed of polyurethane on the polymethyl methacrylate plate above a diode.

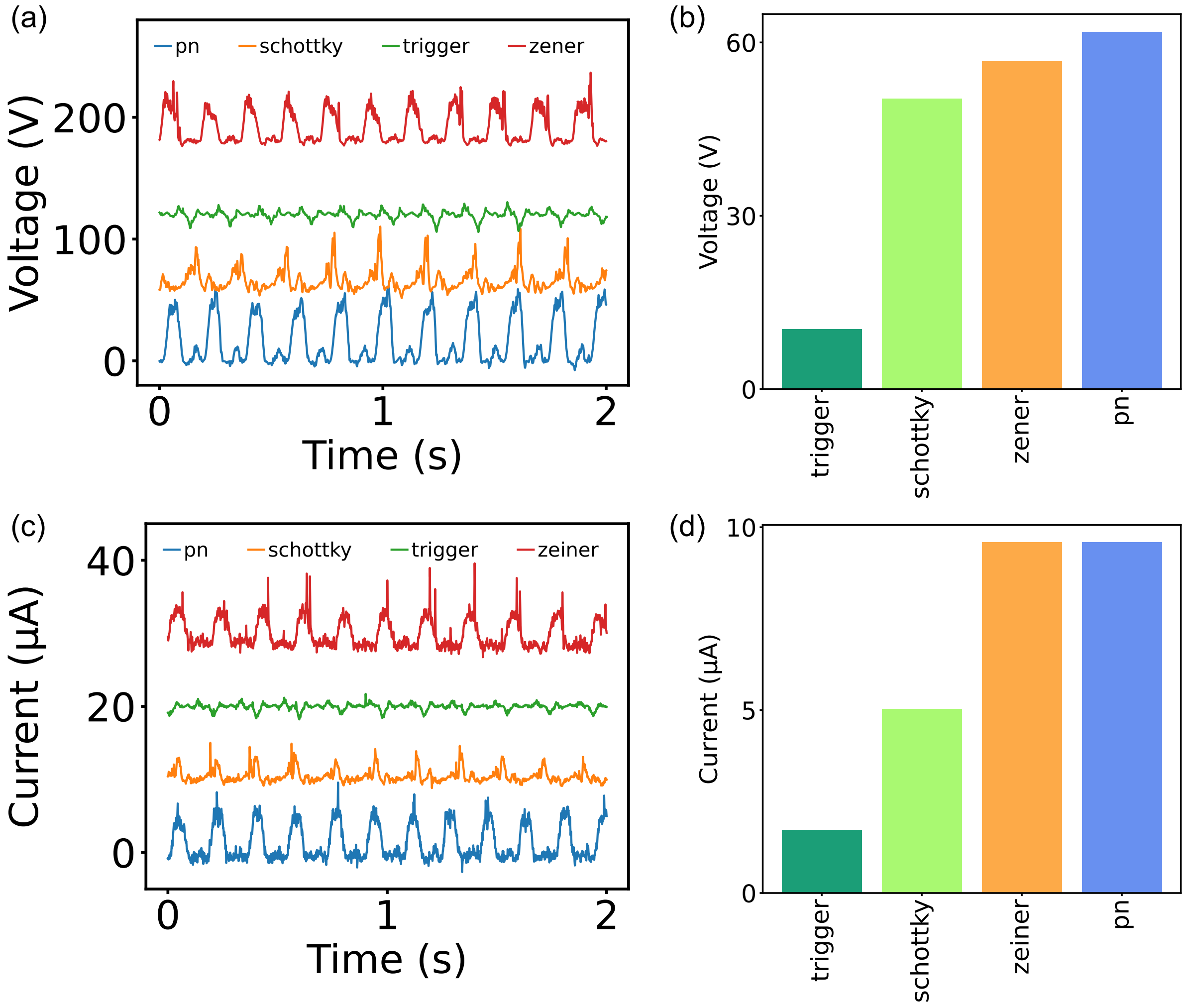

Figure S7. Voltage and current measure on different diode that induced by in-situ generated electrostatic field as in Figures 1h and 1i. (a) A plot of the voltage vs time, (b) A bar plot of the maximum voltage that measured on the diodes. (c) A plot of the current vs time, (d) A bar plot of the maximum current that measured on the diodes.

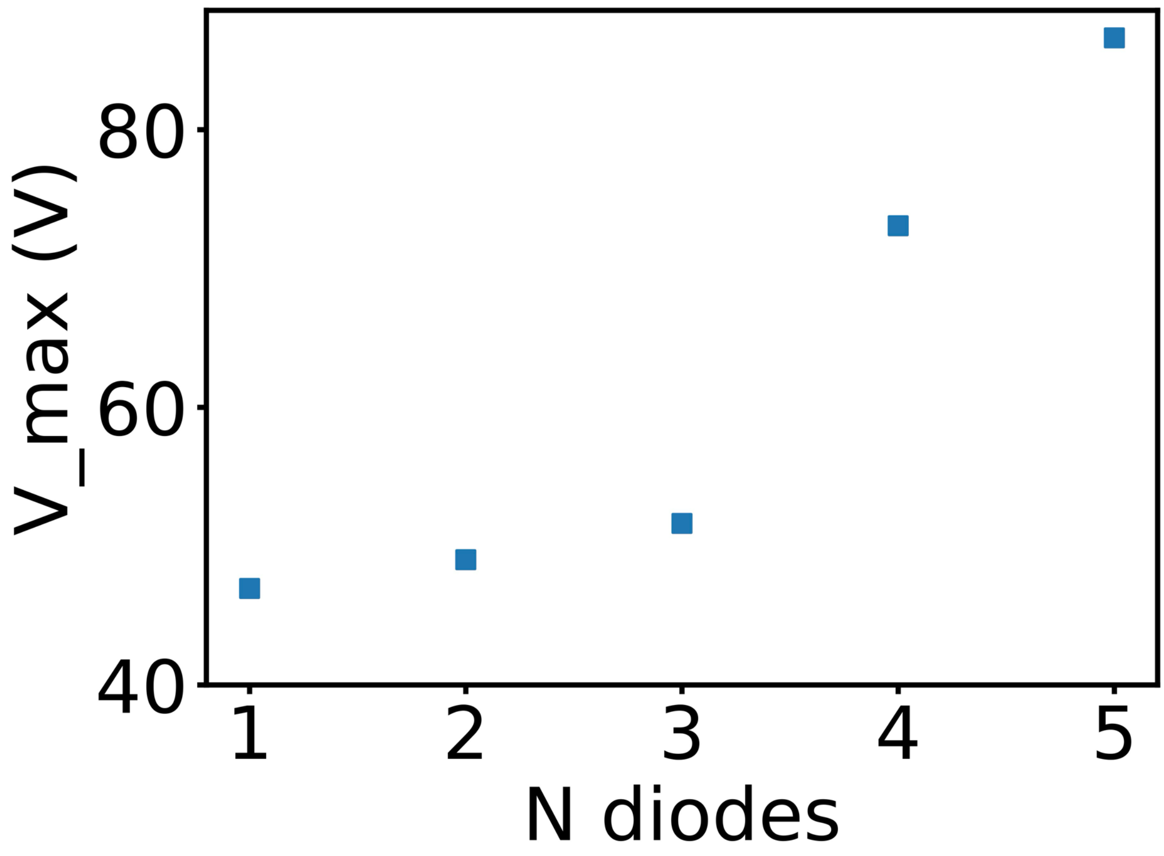

Figure S8. The maximum voltage that measured on DiCes with different numbers of diodes, showing a non-linear relationship.

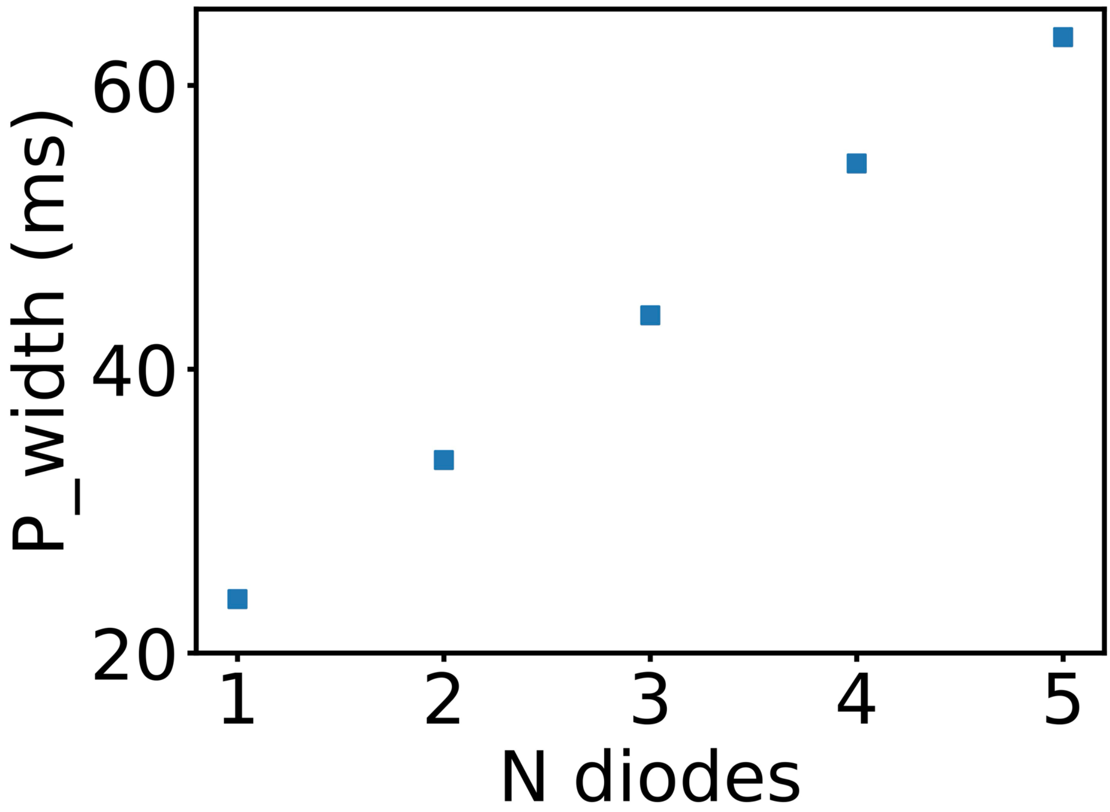

Figure S9. The peak width that measured on the DiCes with different numbers of diodes, indicating a linear relationship. Such a results implies that the energy generation of the DiCe is dependent on the numbers of diodes.

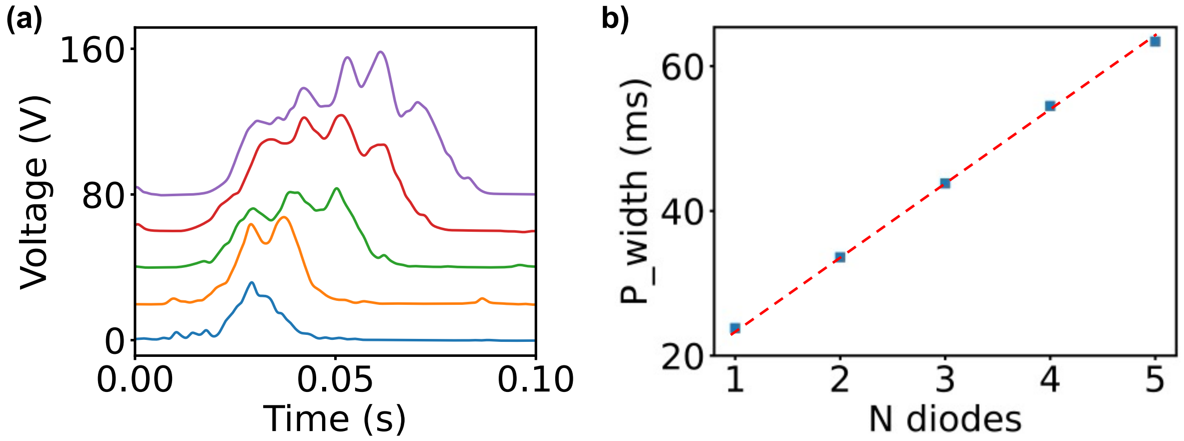

Figure S10. (a) Overlay of voltage signal on diodes. From bottom to top, the number of diodes is 1, 2,3,4, and 5. (b) A plot of the signal width vs the number of diodes.

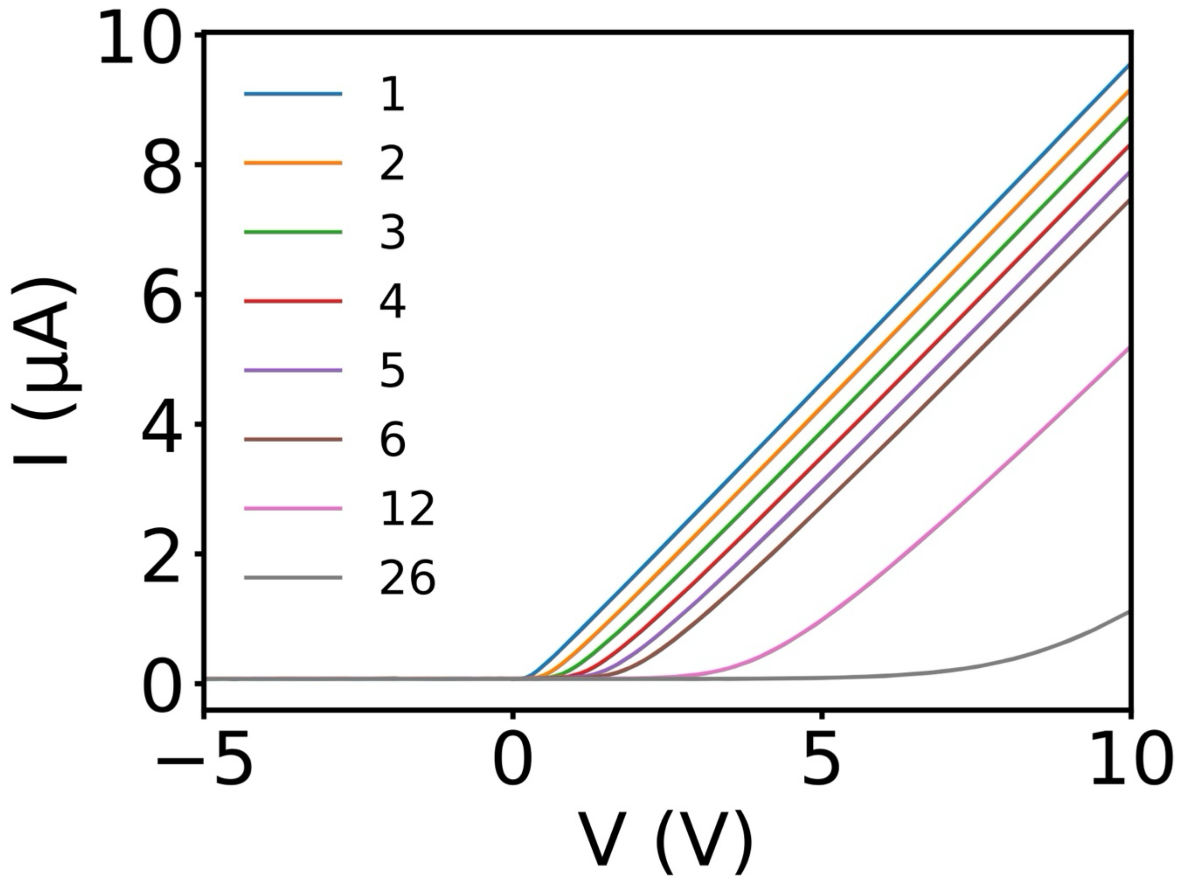

Figure S11. IV characterization of DiCes contains different number of serially connected diodes.

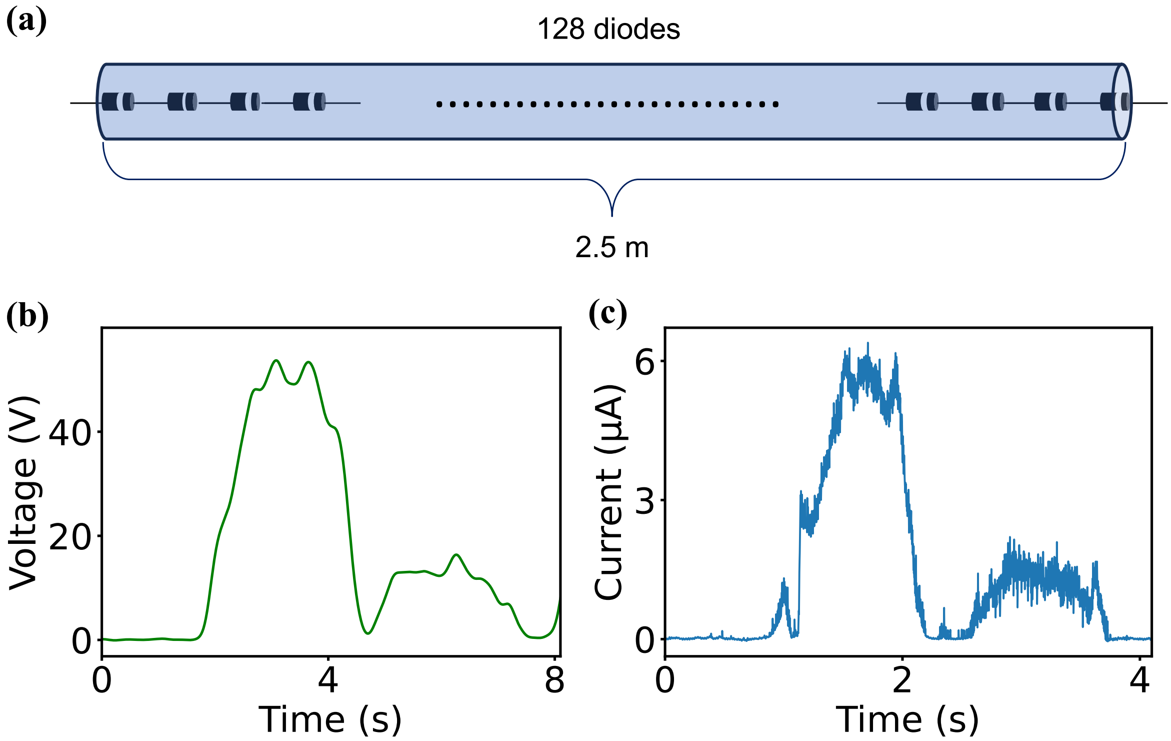

Figure S12. DiCe with 128 diodes in a PTFE tube. (a) A schematic drawing of the structure. (b) The measured voltage on the DiCe while rubbing a cotton on the PTFE tube forth and back. (c) The measured current on the DiCe while rubbing a cotton on the PTFE tube forth and back.

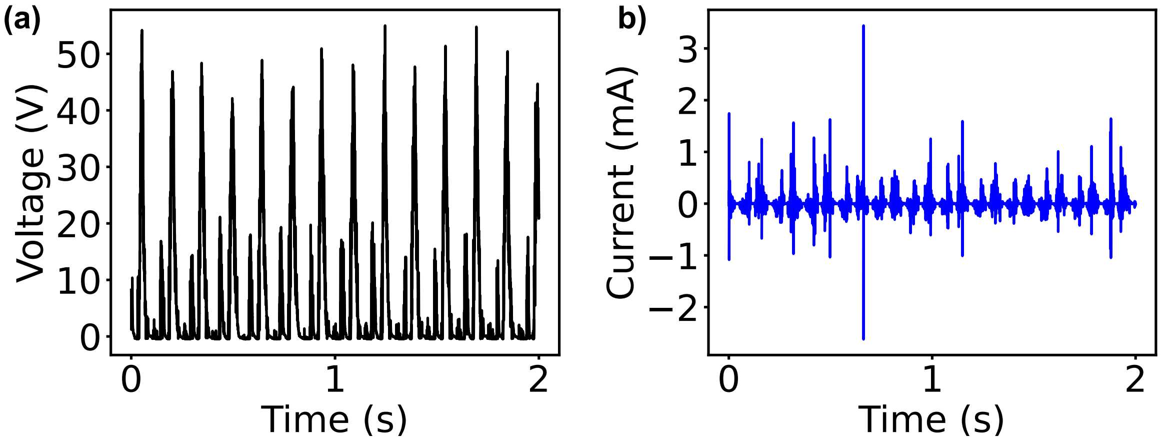

Figure S13. The open circuit voltage (a) and short circuit current that measured on the single diode DiCe that shown in Figures 2i and 2j.

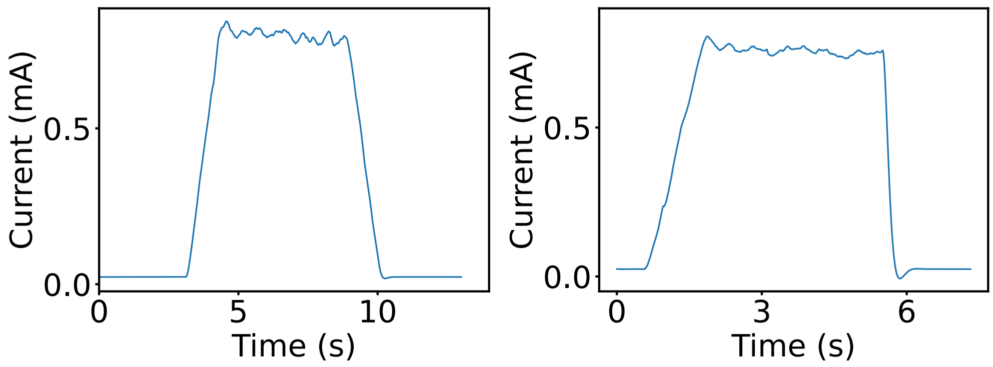

Figure S14. Stability of the DiCe II at the beginning of the test (left) and after 50 000 cycles of rotation. The figures show the measured short circuit current.

Energy recycling on tires and road

DiCes is a type of stationary energy harvesting device that requires no displacement of components as in TENGs[1] and no mechanical work to be done on it. Such a feature makes it very easy to integrate with other objects. DiCes could be mounted inside car tires and under a road to spontaneously harvest energy while a car is running on a road (Figure S15a). Figure S15b shows the electrostatic fields of a simulation of a tire rolling on a road. The potentials of four points inside the tire and nine points under the road are checked after the simulation. Figure S145c plotted the potential of the four points inside the tire while the tire rolled one cycle, indicating there are differences at the four points. Figure S15d shows the difference of potentials to a reference point (w1). Such a difference could drive electron flow over a DiCe that is mounted. Similarly, Figure S15e and Figure S15f plotted the potential of the nine points under the road and the difference between the reference point (r1) versus the position of the tire. The simulation results have shown that it is possible to harvest energy spontaneously from the tire and the road mounted with DiCes.

Experimentally, we mounted a DiCe under a PTFE tape to mimic the road (r-DiCe) and a DiCe inside a tire (t-DiCe), and measured the electrical signal from both of the DiCes to verify the simulation results. Figure S15h and Figure S15i show the open circuit voltage and short circuit current measure on the r-DiCe at different rolling speeds of the tire with 20 kg load on it. DC voltage and current have been observed. The current at the high-frequency domain could reach about 400 μA, while it was sub-μA at the low-frequency domain. To study the energy output, we charged a 1μF capacitor with the r- and t-DiCe at different tire rolling speeds and load weights. Results (Figure S15j~S5m) have shown that the charging speeds are proportional to the speed of the tire rolling. However, a non-linear relationship was observed between the charge speed and the load weight on the r-DiCe (Figure 16).

Energy recycling on tires

Here we try to estimate the energy recycling from the friction between the tires and the road. We used an 18 cm diameter tire as a model and the PTFE taped floor as a model road.

A diode cell containing 28 serially connected diodes was inserted in the tire and the electric output was conducted via a slip ring. A 10 kg load was put on the tire for the test The energy output was measured by charging a 1 μF capacitor while rolling the tire on the model road at different speed.

In order to estimate the energy output from the diode cell that could be mounted on a car tire (195, R55, 16 inches), parameters like the loaded weight, speed, dimension, and diode cell need to be included. Here are the parameters that have been taken:

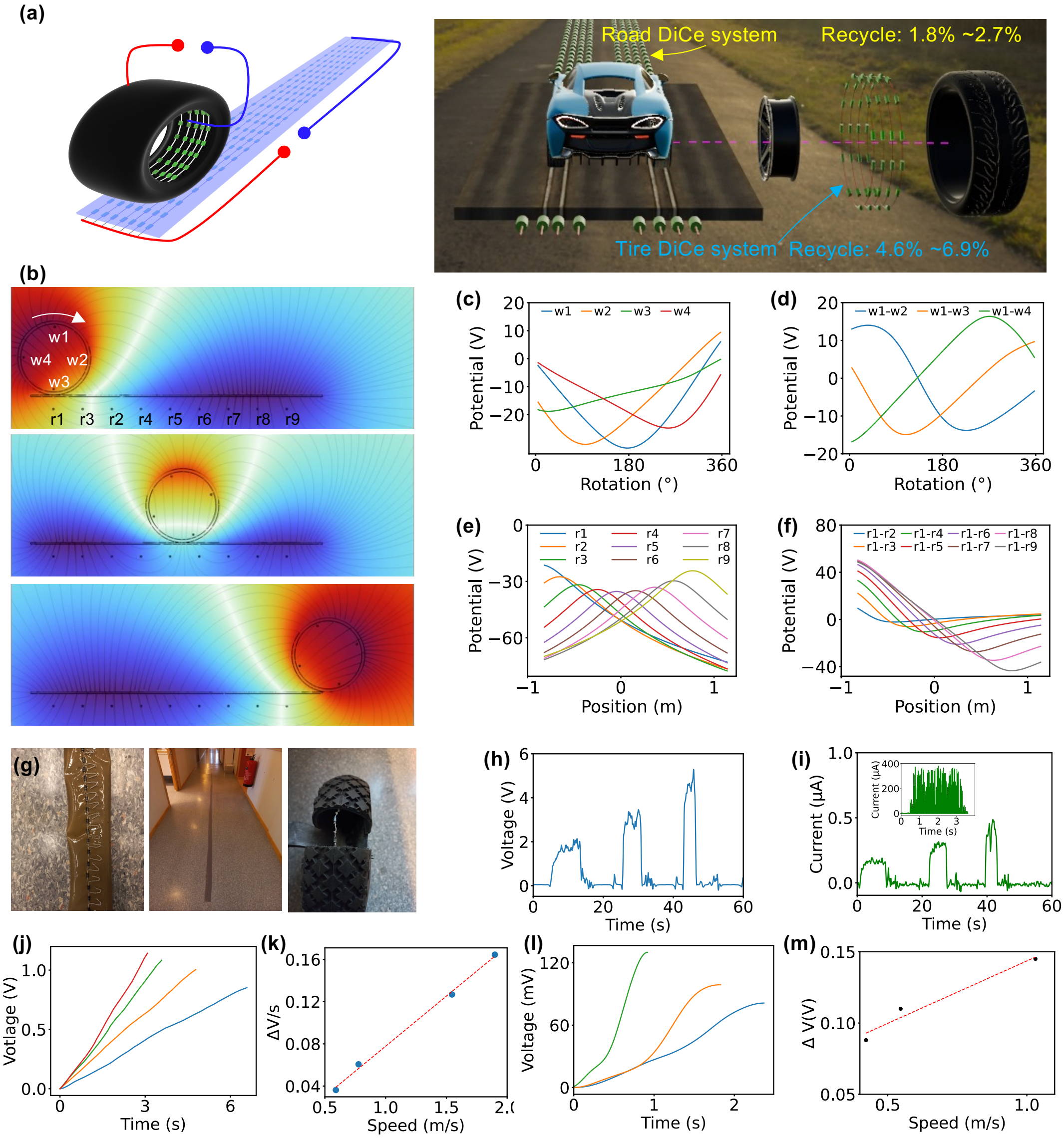

Figure S15. Spontaneous energy harvesting on cars and roads. (a) A schematic drawing of the strategy for harvesting energy from cars and road mounted with DiCes spontaneously, and 3D model of future applications. (b) A COMSOL simulation of a tire rolling on a road. w1~w4 and r1~r9 show the position where electrostatic potentials were measured. (c) The electrostatic potential on the four positions inside the tire, and (d) the potential difference of w2, w3 and w4 to w1. (e) The electrostatic potential on the nine positions on the road, and (f) the potential difference of r2~r9 to r1. (g) A photograph shows the DiCe attached to a PTFE and on the floor for mimic a r-DiCe system, and a photograph of the t-DiCe system where a DiCe is put inside a tire. (h) The open circuit voltage and (i) the short circuit current on the r-DiCe at different tire rolling speed. The insert shows the current before the filtering of high-frequency signal. (j) Charge a 1 μF capacitor by the r-DiCe at different tire rolling speed and (k) the voltage increase per second at different speed. (l) Charge a 1 μF capacitor by the t-DiCe and (m) the voltage increase per second at different tire rolling speed. The 3D model in (a) was created using Blender (Version 4.0.2).

Table S1: Parameters of experiment and the target electric car

**Experimental**		**Target model (Electric car)**	
**Tire size**	Width: 5 cm<br>diameter:18 cm	**Tire dimension**	195/55R16<br>width: 19.5 cm<br>diameter: 62 cm
**Load weight**	10 kg	**Weight**	2000 kg
**Speed**	~6.6 km/h	**Speed**	100 km/h
**Diode cell**	1	**Diode cell**	65/tire x 4 (tires)
**Total diodes**	28	**Total diodes**	100302
**Density of diodes**	1 diode / 2 cm	**Density of diodes**	4 diodes / 2 cm

While we charge the capacitor at different speeds, we have got a linear relationship between the voltage increase (ΔV) per second on the capacitor and the speed (v) that is

$$\Delta V = 0.095 * v - 0.017 \quad (1)$$

Therefore, the ΔV will be 2.62 V/s at a speed of 100 km/h. **Ideally**, a 1 μF capacitor will be charged to 9438 V after 1 hour, which equals an energy storage of 44.54 J.

If the weight effect has been included (**assume the co-efficient factor of the weight and the speed is 1**), the energy storage would be 110 J, according to the nonlinear relationship between voltage increase (ΔV) per second and the loaded weight (W).

$$\Delta V = 290.65907 * W^{0.0002242591} - 290.64098 \quad (2)$$

According to this relationship, the energy output at a load of 500 kg is 3 times the load of 10 kg.

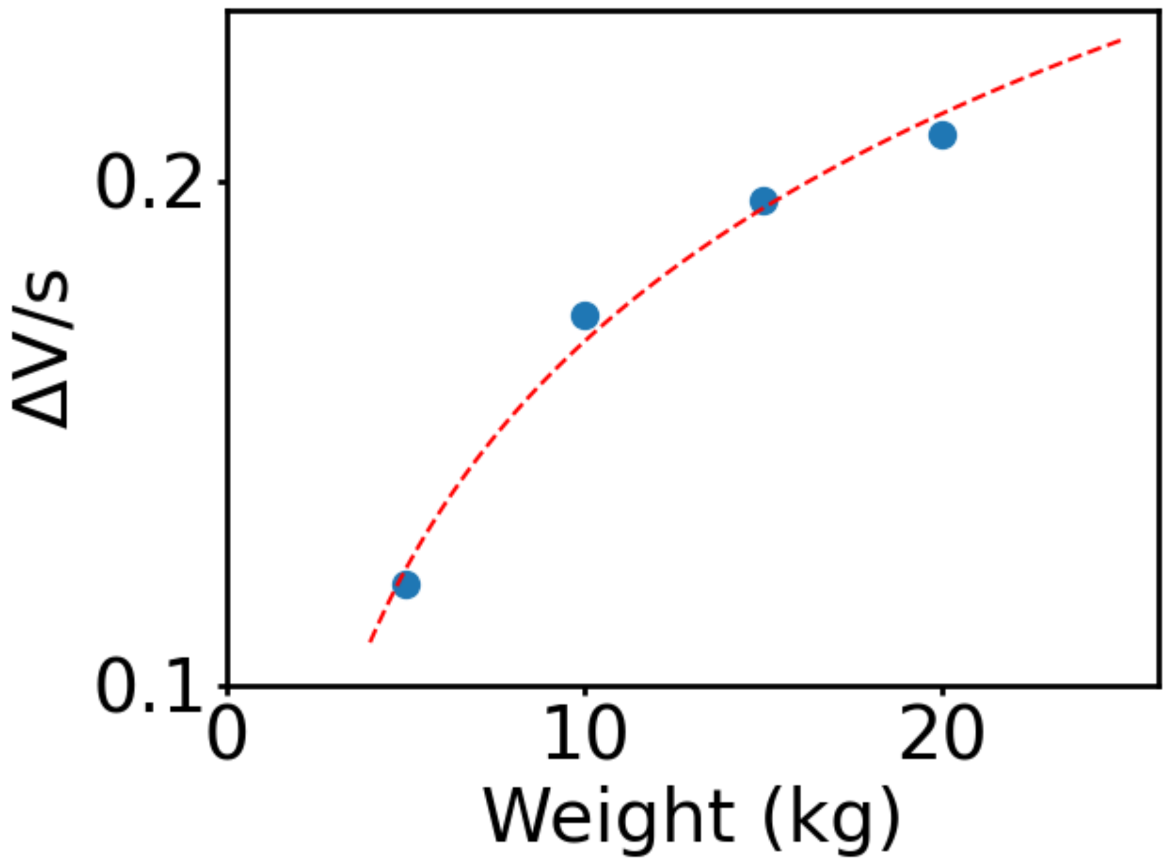

Figure S16. A plot of the charging speed of a 1 μF by the r-DiCe at different loaded weight.

At the target model, there will be more diode cells to be installed which is calculated ideally at 65 diode cells per tire. Moreover, the density of diodes at unit length could be 4 times of the test one. The contact area of the car tire is estimated 10 times of the wheel used in the experiment. However, since the number of diode cells included the impact of the width of contact patch, the factor from the change in contact area is estimated as 2.5 times. In our experiment, we have found that the energy output of diode cells is linearly related to the length. We assume here the output is also linearly related to the density. Based on this experimental data and assumptions, we can estimate a tire run at a speed of 100 km/h for 1 hour could lead to energy output to a capacitor is:

$$110 * 65 * 4 * 2.5 = 71500\,J$$

Therefore, a car could produce 286000 J per hour with diode cells mounted in four tires. If we take advantage of the volume effect that has been experimentally proved, the output could be doubled to 572000 J which equals 159 Wh. Such a number means that, ideally, we can harvest 159 Wh electricity from the friction between the tire and the road based on the DiCes in the car tires.

For the target car model which is an electric car, the energy loss to the tire rolling resistance is about 23% of the total energy consumption[2]. An electric car usually has an energy consumption

of 10 to 15 kWh. Therefore, the energy loss on friction is 2.3 to 3.45 kWh. Taking this number and the number from our estimation, we could ideally recycle 4.6% to 6.9 % percent of the energy consumption of the tire rolling resistance, that is 1.2% to 1.6% of the total energy consumption of an electric car.

Energy recycling on road

Besides the installation in car tires, diode cells are also compatible to be integrated into road structures. In our experiment, we used PTFE taped floor as a road model in order to explore the up limit of the energy recycling on the road while the car is running.

While we charge the capacitor at different speeds, we have also got a linear relationship between the voltage increase (ΔV) per second on the capacitor and the speed (v) that is

$$\Delta V = 0.109 * v - 0.05 \quad (3)$$

Which gives an energy output of 57.1 J per hour at a speed of 100 km/h. Taking the same weight effect, the energy output would be 171 J.

The number of diode cells could be the same to that installed in a tire, that is 65. The density of diodes per unit length is 2 times as the diode cells put under PTFE have a higher density than that in the tire. The contact area effect is 2.5 that is the same for calculating energy output from the tires. With this data, we can have

$$171 * 65 * 2 * 2.5 = 55575\,J$$

Energy output from diode cells on a road, which equals 15.4 Wh per tire. That gives a total output of 61.6 Wh with a car that rolling on the road.

If we include both the energy harvest from the tire and the road, we can get 221 Wh energy output in total. That said, we could ideally recycle 6.4% to 9.6% of the energy loss due to the rolling resistance of tire.

Discussion on the parameters

The estimated energy recycling of the ideal case (supplementary information.) has shown a promising potential to reduce the energy consumption of cars. Compared to the kinetic energy recovery systems (KERS)[3] that have high output power up to tens of kW, the power of the DiCe based system is relatively low. However, if one looks at the energy output, the two systems are on the same level. A KERS can have a power of 60 kW for 6.67 seconds[4], equalling 111 Wh energy. Such a number is close to the estimated energy output from the DiCe system at an ideal condition. However, the actual output from the DiCe system may be lower than the estimation, requiring more multidisciplinary studies.

In this estimation, we have the weight, speed and density effect to the energy output established based on the experimental results. However, such effects are experimentally proved separately. Therefore the synergistic effect of these parameters is an uncertainty that needs further studies.

A co-efficient factor that smaller than 1 would be expected. The impact of contact area was taken as a linearly relevant parameter which may create some percentage of error.

In real cases, the tire is rolling on asphalt that has a different contact electrification property to PTFE. Therefore, the real effect may be low than we have estimated here using PTFE. However, we still have a chance to recycle about 1 % of the energy loss even if the contact electrification in the real case is one-tenth of our estimation.

Unique features of the DiCes

Free motion in space and on surfaces

Electrostatic induction serves as the fundamental physical principle underpinning DiCes, which is a concept also embraced in TENGs. For TENGs, the efficacy relies heavily on the meticulous alignment of motion vectors and device architectures. However, such constraints do not limit the operational versatility of DiCes. Figure S17a illustrates seven distinct motions (Figure S17b) that capable of inducing electricity generation within a single-diode DiCe. Results illuminate that unrestricted motions of a charged object in the space surrounding a DiCe proficiently convert mechanical energy into electricity. Three motions were specifically chosen to charge a 1μF capacitor (Figure S17c), demonstrating the efficacy of energy harvesting. Another demonstration is given in the supplementary information where a LED-DiCe was fabricated and encapsulated with FEP a film. A rubbing or touching of a finger on any of the six sides of the DiCe could lit up the LEDs (Supplementary Video S5), These two experiments demonstrated that a DiCe is not sensitive in operating directions, allowing free motion in space.

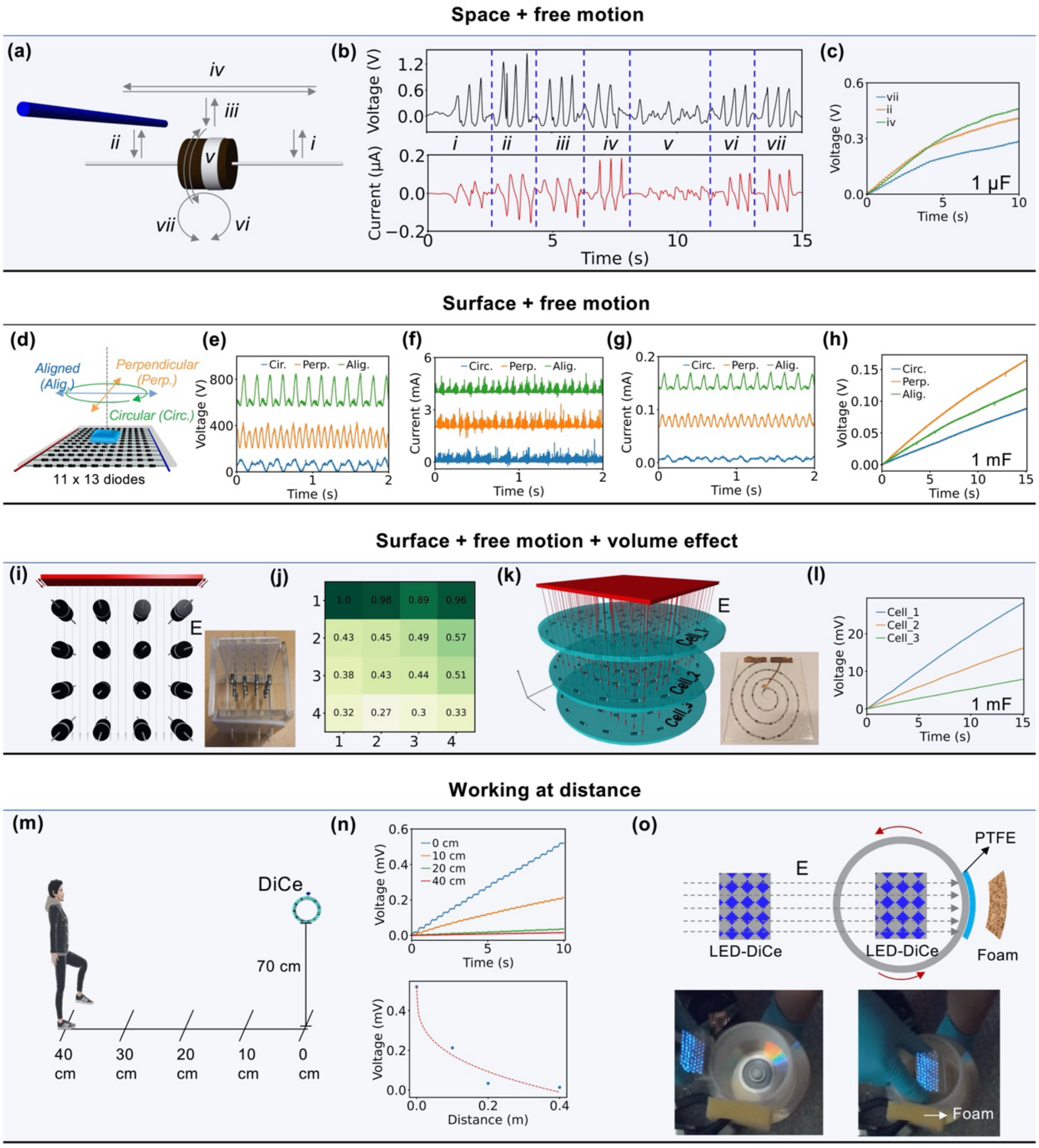

Figure S17. Unique features of the DiCe. (a) ~(c) free motion in the space of a charged rod. (a) A schematic drawing shows 7 motions around a DiCe. (b) Open circuit voltage and short circuit current measured on the DiCe while doing motions. (c) Charge generation on the DiCe by selected motions. (d)~(h) free motion on a surface of a DiCe. (d) A schematic drawing of the structure of the DiCe and the operation modes. (e) Open circuit voltage, short circuit current before (f) and after (g) filtering of high frequency signals, measured on the DiCe (11 x 13 diodes). (h) Charge generation on the DiCe by the three motions. (i) ~(l) Energy harvesting with volume effect. (i) A scheme of a 4 x 4 array of 16 diodes with a charge rod that moves above, and a photograph of the construction. (j) A heatmap shows the relative electrical intensity of each diode to diode (1,1). (k) A scheme of three DiCes (serially connected 28 diodes rolled as a spiral) and a photograph of a DiCe that used. The surface of the top DiCe was cover by FEP tape, and a piece of cotton was used to generate an onsite electrostatic field. (l) Voltage that was measured on a 1 mF capacitor charged by the DiCes. (m) and (n) Energy harvesting at different distances. (m) A scheme drawing shows a person stepping at different distances to a DiCe. (n) Charge a 1 μF capacitor at different distances for 10s. (n) A plot of the voltage on

the capacitor versus the distance. (o) Powering of a LED-DiCe at distance, where the LED DiCe was place either inside or outside of a rotating cylinder. A piece of PTFE was attached to the outside of the cylinder to generate charges on its surface with a polyurethane foam.

A DiCe can also be operated analogously to TENGs by sliding a dielectric material over it, distinct from TENGs where the slider typically moves back-and-forth in a singular direction. In contrast, a DiCe slider can move freely on the counter dielectric material's surface, exemplified in Figure S17d, where a piece of cotton rubs on a PTFE-covered DiCe (11 x 13 diodes). Movements of the cotton can be aligned, perpendicular to the direction of the diode connection, or circular on the surface. DC-like voltages (Figure S17e) and current (Figure S17f, S17g) were observed on the DiCe when operated through the three motions. Figure S17h illustrates the result of charging a 1 mF capacitor with the DiCe subjected to different movements. Remarkably, even without circuit optimization, the charge stored in the capacitor after 15 s could exceed 0.15 V (Figure S17h), closely approaching the state-of-the-art output from TENGs with optimized power management[5]. Beside the construction of the DiCe presented in Figure S17d, we have tested two other constructions that allow free surface motions (Figures S18 and S19).

Volume effect: Beyond the discussed free motions in space and on surfaces, DiCes exhibit the capability of being stacked to harvest energy from a dynamically changing electrostatic field, showcasing a volume effect (Figure S17i~17l). To demonstrate this effect, a 4 x 4 diode array was constructed, and the voltage across the diode was measured while exposed to a moving charged PVC tube above the array. Figure S17jpresents a heatmap of the normalized voltage measured on each diode (Figure S20), indicating reduced charging generation with increased distance from the electrostatic field. To further demonstrate the volume effect, three DiCes were fabricated and stacked on top of each other (Figure S17k). By rubbing a piece of cotton on a PTFE film covering the top DiCe, charges were generated on the three cells and stored by charging a 1 mF capacitor. Results (Figure S17l) reveal that the total charges stored by all three DiCes were twice that using only one DiCe, showing an obvious volume effect.

Working at distance: Another unique feature of the DiCe is that it can harvest energy at a distance to a charged moving object, taking advantage of the spread of the electrostatic field over space and distance. In our experiment, a 1 μF capacitor was charged up by a DiCe put in front of a person who was stepping on the floor (Figure S17m). The capacitor has been charged to 0.21 mV at a distance of 0.2 m compared to 0.52 mV where the DiCe was put in the pocket (Figure S17n). A nonlinear relationship between the voltage and the distance was observed. Another demonstration was done by put a LED-DiCe (See circuit in Figure S21) inside or outside a rotating cylinder. On the outside wall of the cylinder, a piece of PTFE tape was attached on it, and a piece of polyurethane foam was used to created triboelectrification with the PTFE while the cylinder is rotating (Figure S17o). Results have shown that the LEDs were lit up both inside and outside the cylinder, showcasing the energy harvesting at distance.

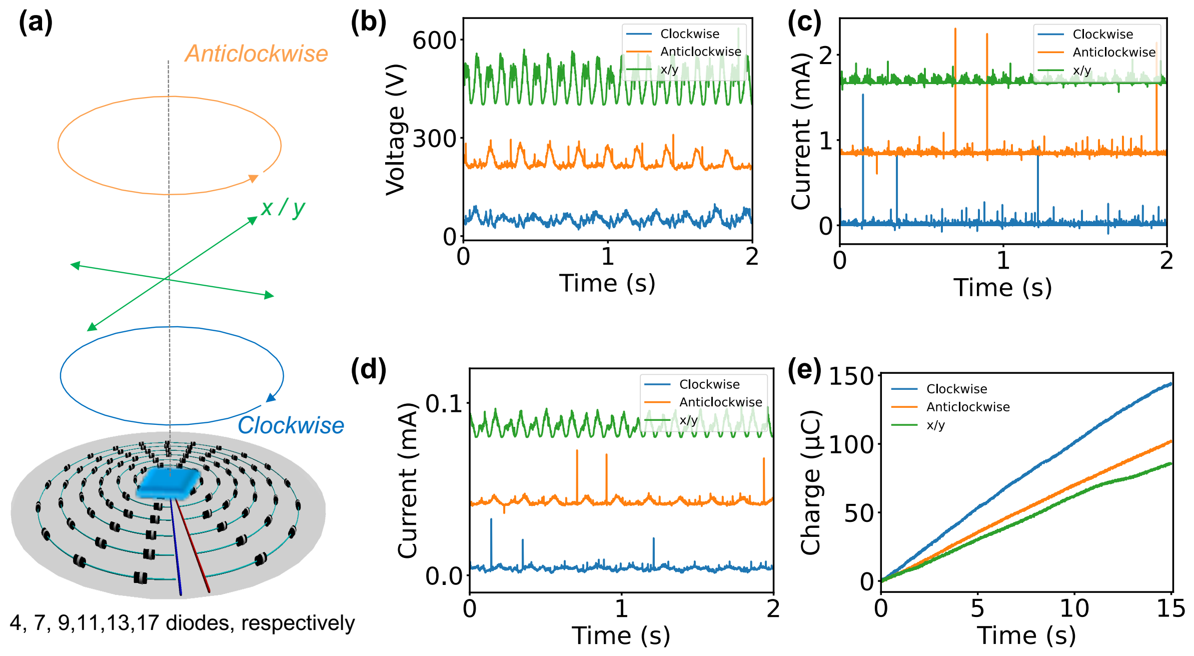

Figure S18. Free motion on the surface of a DiCe, where the diodes in the circles are serially connect and the circles are parallelly connected. (a) A schematic drawing of the DiCe structure and the movements of a cotton on the surface. (b) Open circuit voltage, short circuit current before (c) and after (d) filtering of high frequency signals, measured on the DiCe. (e) Charge generation on the DiCe by the three motions.

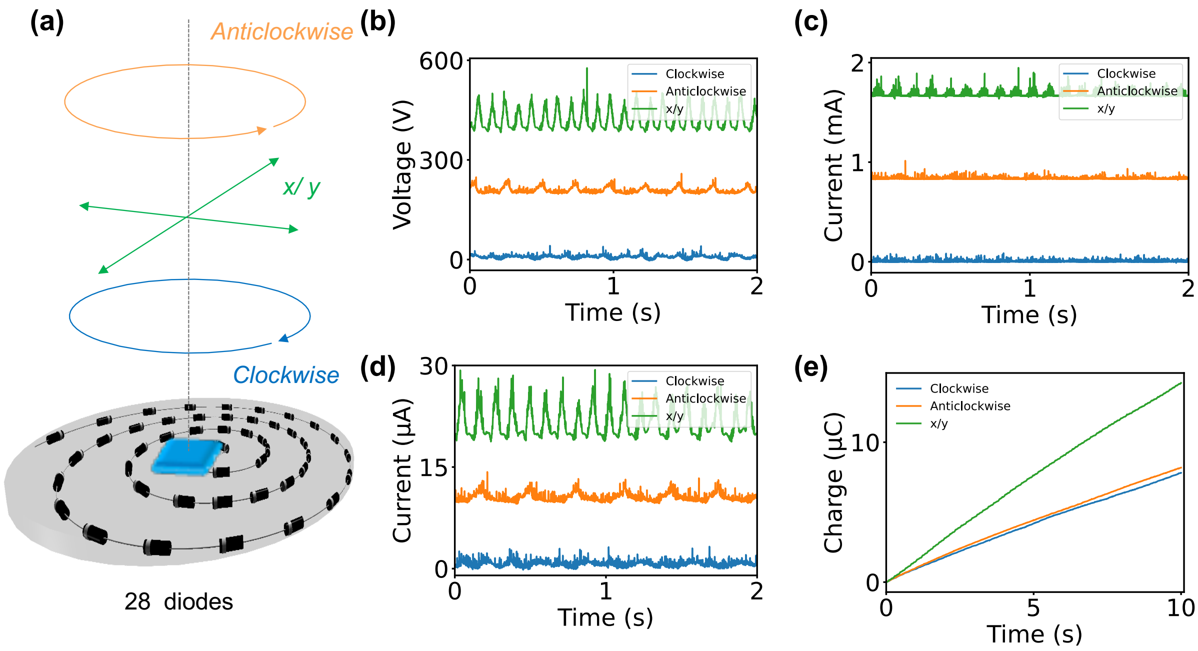

Figure S19. Free motion on the surface of a spiral DiCe. (a) A schematic drawing of the DiCe structure and the movements of a cotton on the surface. (b) Open circuit voltage, short circuit current before (c) and after (d) filtering of high frequency signals, measured on the DiCe. (e) Charge generation on the DiCe by the three motions.

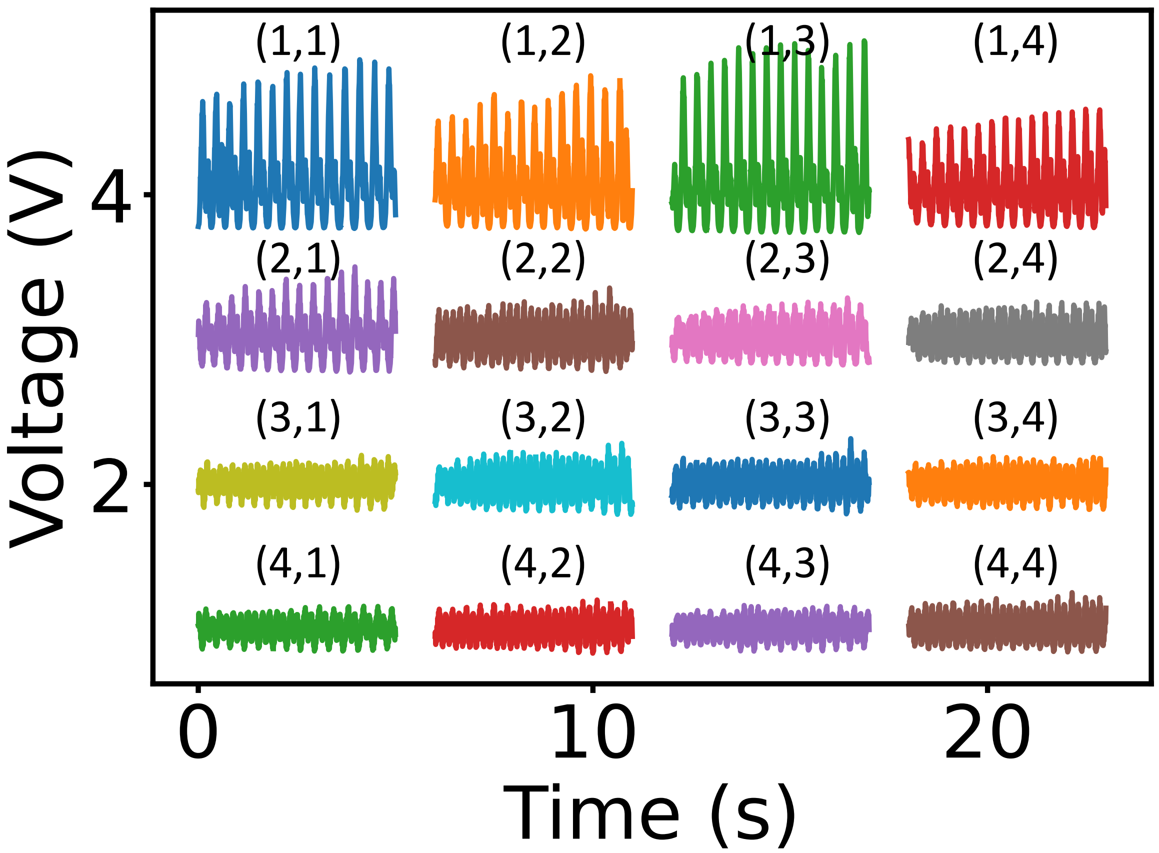

Figure S20. Voltage measured on each diode of the 4 x 4 array that described in Figures 3i and 3j.

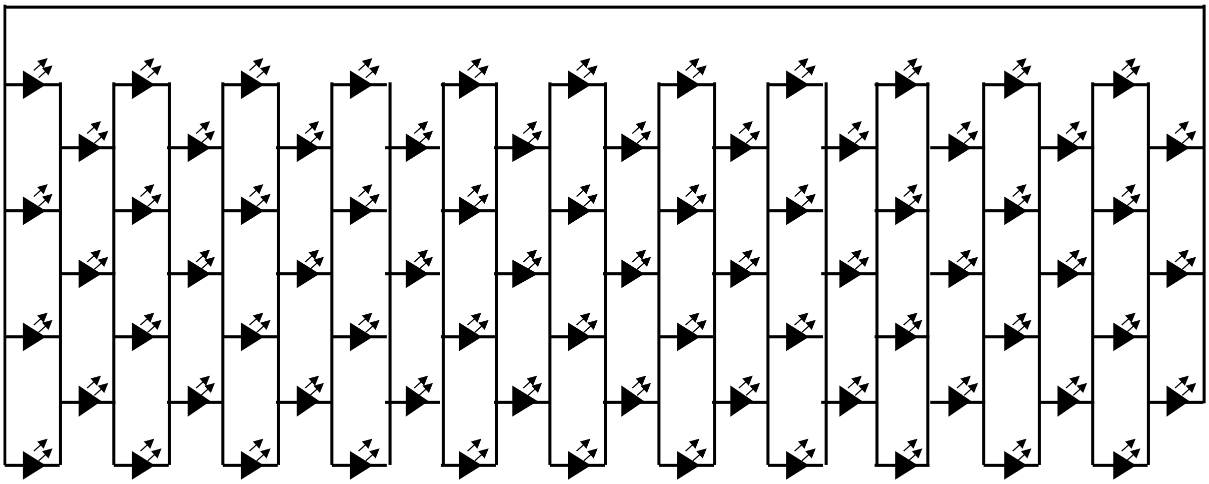

Figure S21. The circuit of the LED-DiCe.

Table S2. Comparison of DiCe and TENG/PENG Devices in articles published in the past two years.

Device		Peak Output Voltage (V)	Peak Output Current (μA)	Power Density (μW/cm^2)
This work	DiCe-I	Up to 709	68600	243187
	DiCe-II (**DC**)	Up to 490	1080	2650
TENG[6]		1717.7	129	380
TENG[7]		400	0.4	2 (μW)
PENG[8]		12.2	-	14.85
PENG[9]		12.2	1300	42.5
Hybrid TENG/PENG [10]		52.3	1.23	8.5
TENG [11]		5650	18	832
TENG [12] (**DC**)		4065	27.5	877
TENG [13]		745	22.5	760
TENG [14]		39.8	4.38	23
TENG [15]		261	1.5	65.4
TENG [16]		133.8	21.9	53
PENG[17]		200	16.3	1132
TENG[18]		1485	4.1	6.25
TENG[19]		2580	41	67.26 (μW/cm^3)

References:

1. Cao, X. *et al.* Inductor-Free Wireless Energy Delivery via Maxwell's Displacement Current from an Electrodeless Triboelectric Nanogenerator. *Advanced Materials* 30, 1704077 (2018).
2. Where the Energy Goes: Electric Cars. https://www.fueleconomy.gov/feg/atv-ev.shtml.
3. Farrokhzad Ershad, N., Tafazzoli Mehrjardi, R. & Ehsani, M. High-Performance 4WD Electric Powertrain With Flywheel Kinetic Energy Recovery. *IEEE Trans Power Electron* 36, 772–784 (2021).
4. Wikipedia contributors. Kinetic energy recovery system. *https://en.wikipedia.org/wiki/Kinetic_energy_recovery_system#:~:text=A%20kinetic%20energy%20recovery%20system,for%20later%20use%20under%20acceleration.*

5. Liu, W. *et al.* Switched-capacitor-convertors based on fractal design for output power management of triboelectric nanogenerator. *Nat Commun* 11, 1883 (2020).

6. Cho, W. *et al.* High-Performance Yet Sustainable Triboelectric Nanogenerator Based on Sulfur-Rich Polymer Composite with MXene Segregated Structure. *Advanced Materials* 36, 2404163 (2024).

7. Li, W., Lu, L., Zhang, C., Loos, K. & Pei, Y. Durable and High-Performance Triboelectric Nanogenerator Based on an Inorganic Triboelectric Pair of Diamond-Like-Carbon and Glass. *Advanced Science* 11, 2309170 (2024).

8. Sahoo, S. *et al.* High-Performance Piezoelectric Nanogenerator and Self-Charging Photo Power Cell Using Hexagonal Boron Nitride Nanoflakes and PVDF Composite; High-Performance Piezoelectric Nanogenerator and Self-Charging Photo Power Cell Using Hexagonal Boron Nitride Nanoflakes and PVDF Composite. (2024) doi:10.1002/adem.202400658.

9. Prajesh, N. *et al.* Flexible Piezoelectric Nanogenerator with a Ferroelectric Metal-Ligand Cage for Self-Powered Sensor Applications. *ACS Appl Energy Mater* (2025) doi:10.1021/ACSAEM.5C00269/ASSET/IMAGES/LARGE/AE5C00269_0006.JPEG.

10. Das, N. K. & Badhulika, S. Flexible Tribo-Enhanced Piezoelectric Nanogenerator Based on Aluminium Ferrite Electrospun Hybrid Nanofibers for Energy Harvesting and Patient Rehabilitation Application. *Advanced Sensor Research* 3, 2400023 (2024).

11. Liu, B. *et al.* Improving contact efficiency by novel powder forming film for high output DC-triboelectric nanogenerator. *Nano Energy* 130, 110094 (2024).

12. Li, Q. *et al.* A Robust Constant–Voltage DC Triboelectric Nanogenerator Using the Ternary Dielectric Triboelectrification Effect. *Adv Energy Mater* 13, 2202921 (2023).

13. Wang, Q. *et al.* Nature-inspired scalable high-performance triboelectric nanogenerators for energy harvesting and sensing. *Nano Energy* 121, 109217 (2024).

14. Baburaj, A. *et al.* High-performance biodegradable triboelectric nanogenerators based on hydroxypropyl methylcellulose and zinc oxide hybrid composites. *Nano Energy* 128, 109943 (2024).

15. Zheng, Z. *et al.* High-Performance All-Textile Triboelectric Nanogenerator toward Intelligent Sports Sensing and Biomechanical Energy Harvesting. *ACS Appl Mater Interfaces* 16, 10746–10755 (2024).

16. Yan, J. *et al.* High-performance textile-based triboelectric nanogenerators with damage insensitivity and shape tailorability. *Nano Energy* 126, 109675 (2024).

17. Jiao, Y. *et al.* High-performance triboelectric nanogenerators based on blade-coating lead halide perovskite film and electrospinning PVDF/graphene nanofiber. *Chemical Engineering Journal* 483, 149442 (2024).

18. Das, J. P. *et al.* From Friction to Function: A High-Voltage Sliding Triboelectric Nanogenerator for Highly Efficient Energy Autonomous IoTs and Self-Powered Actuation. *Small* 20, 2405792 (2024).

19. Su, E. *et al.* Buoyancy-gravity optimized triboelectric nanogenerators via conductive 3D printing for robust wave energy harvesting. *Materials Science and Engineering: R: Reports* 164, 100953 (2025).
